# The nature and sources of international variation in formal institutions related to initial coin offerings: preliminary findings and a research agenda

**DOI:** 10.1186/s40854-022-00405-x

**Published:** 2023-01-06

**Authors:** Nir Kshetri

**Affiliations:** The University of North Carolina at Greensboro, Greensboro, USA

**Keywords:** Blockchain, Cryptocurrencies, Crypto-ventures, Initial coin offerings, Regulatory sandboxes, Tax havens, F52, G15, G28, O31, O38

## Abstract

As prior researchers have suggested, a firm’s success in an international market depends on how well its strategy fits the nonmarket environment, such as formal institutions. This paper examines the determinants of formal institutions around new areas of economic activities. Specifically, we propose a framework for understanding how the quality of formal institutions in promoting entrepreneurship drives the focus of such institutions concerning initial coin offering (ICO), which is emerging as a popular fundraising method. The paper uses inductive analysis to examine how nonmarket factors—such as a jurisdiction’s tax haven nature, regulators’ perceptions of ICOs as threats to national or political interests, and trade and industry associations—might moderate the relationship between the quality of institutions and the focus of such institutions regarding ICOs. One of this study’s key findings is that an economy’s quality of entrepreneurship-related institutions, perceived threats to national/political interests, and tax haven nature lead to different policy orientations. Consequently, regulators assign different importance when promoting crypto-entrepreneurship and dealing with associated risks. Regulators focusing mainly on promoting crypto-ventures have taken measures to enrich the blockchain ecosystem and provided tax and non-tax incentives to attract such ventures. Regulators focusing mainly on dealing with crypto-venture risks rely on a regulatory sandbox and close regulatory monitoring of such ventures.

## Introduction

Prior research in a range of different contexts, such as the circular economy (Khan et al. 2021a, b; Yu et al. 2022), property registry (Kshetri 2022), farm insurance (Kshetri 2021), and supply chain sustainability (Khan et al. 2021c) has suggested that blockchain has diverse economic and social benefits. Raising funds for entrepreneurial ventures is among the key use cases of blockchain technology (Xu et al. 2019). In this regard, a fascinating, rapidly evolving phenomenon facilitated by blockchain is a capital-raising method known as initial coin offering (ICO), which involves offers and sales of crypto-tokens using blockchain technology (Campino et al. 2022). In 2018, startups raised about 11.4 billion US dollars (USD) worldwide through ICOs (Pozzi 2019). Following the peak level in 2018, the ICO market declined (van Oosterhout 2021). Prior researchers have identified ICO regulations as a key area of concern and challenges that have hindered the growth of the ICO market (de Andrés et al. 2022; Karpenko et al. 2021). For instance, Knott et al. (2022) found that legal and regulatory shortcomings are among artists’ major barrier in raising funds through ICOs.

At a broader level, nations vary widely in terms of what institutional theorists refer to as “rules of the game” (North 1990) and “rule setting, monitoring, and sanctioning activities” (Scott 1995: 42) related to ICOs. For instance, while countries like China (Stanley 2017c) and South Korea (O’Leary 2017b) have imposed strict regulatory measures, such as a total ban on ICOs, others such aslike Puerto Rico (Bowles 2018) and Panama (FTNS 2016) have shown a high degree of openness to ICOs.

Researchers have detailed a variety of policy responses and their effects on different types of financing and investment mechanisms and tools, such as foreign direct investments (FDI) (Blomström et al. 2003; Mallampally and Sauvant 1999), venture capital (VC) (Cumming et al. 2017; Keuschnigg and Nielsen 2003; Da Rin et al. 2006), equity crowdfunding (ECF) (Kshetri 2015), and sovereign wealth funds (SWFs) (Johan et al. 2013; Murtinu and Scalera 2016; El–Kharouf et al. 2010; Drezner 2008). As blockchains are disruptive (WEF 2015), there are various reasons to believe that theories concerning other financing and investment mechanisms may not apply to ICOs. First, prior research has shown that information and communication technologies (ICTs) are key enablers and facilitators in creating new ventures (von Briel et al. 2018); however, blockchain has fundamentally transformed how such ventures are created. This technology allows startups to seek new ways of raising funds beyond their reliance on traditional sources. For instance, prior research has suggested that authoritarian regimes oppose decentralized fundraising, such as crowdfunding (CF) (Kshetri 2015); however, unlike CF, blockchain allows an entrepreneur to reach directly to investors without the need offor ECF platforms. Due to this feature, ICOs may elicit different regulatory responses across various jurisdictions.

Second, anonymity and irreversibility of transactions can fund entities owned or controlled by terrorists (Brill and Keene 2014). Countries differ in their concerns about this issue, which may lead to different regulatory responses to ICOs.

Third, discontinuous and disruptive technologies, such as blockchain and cryptocurrencies, generate exogenous shocks. For instance, in June 2021, more than 1,100 people were arrested in China for allegedly using cryptocurrencies to launder their earnings from online scams (Yu and Ostrof 2021). In these situations, it is difficult for nations to develop guidelines and templates (Greenwood and Hinings 1993) to deal with such technologies. Countries likely differ in the degrees of motivation and flexibility in establishing regulatory and policy guidelines related to ICOs. For instance**,** whereas small countries with a homogeneous population, such as tax havens, exhibit higher flexibility to respond (Read 2001), larger economies with more established institutions related to entrepreneurship may lack such flexibility. Researchers have called for an investigation of regulations affecting cryptocurrency and the ICO market (Xu et al. 2019).

Thus, we need new theoretical approaches to explain formal institutions’ interaction with ICOs’ unique features. This paper aims to fill the gaps in the sparse literature on ICOs and help relevant stakeholders (e.g., regulatory authorities, entrepreneurs, investors, trade associations, etc.) make timely and informed decisions. Specifically, it examines the following research questions: RQ1) What is the nature of divergence in ICO-related formal institutions? RQ2) Are specific economic and institutional characteristics linked to crypto-venture policy responses?

Our framework and analysis contribute to financial innovations and international business literature, especially research in international entrepreneurship. First, we provide a framework for understanding how the quality of formal institutions to promote entrepreneurship drives the focus of such institutions concerning crypto-ventures. Specifically, we explain how economies with higher-quality entrepreneurship-related institutions are more likely to focus on assessing, analyzing, and controlling risks associated with crypto-ventures than economies with lower-quality entrepreneurship-related institutions. Conversely, economies with lower-quality entrepreneurship-related institutions are more likely to focus on promoting entrepreneurial activities in the crypto-arena than economies with higher-quality entrepreneurship-related institutions. In this way, this paper’s framework contributes important insights into factors that affect how the rules of the game (North 1990)—and those monitoring and sanctioning activities (Scott 1995)—evolve around crypto-ventures in economies across different levels of entrepreneurship-related institutions.

The second contribution of our framework is that it highlights how factors related to nonmarket environments, such as trade and industry associations, perceived threats to national and political interests, and the tax haven nature of jurisdiction, moderate the relationship between the quality of institutions and the focus of institutions. The impacts of formal institutions’ quality on the focus of institutions concerning crypto-ventures vary with the levels of these moderators. For instance, trade associations can help develop crypto-accommodating legislation and more favorable game rules for crypto-ventures through framing, justification, and persuasion. In this way, they can stimulate the development of formal institutions to promote entrepreneurial activities in this arena. Specifically, this paper analyzes how the state’s coercive (Groenewegen and Van der Steen 2007) and expert power of trade associations (Kshetri and Dholakia 2009) can be combined to explicate an optimal game rule around new economic activities.

Third, our study highlights the critical role of legal clarity of crypto-tokens and measures to enrich the blockchain ecosystem to promote entrepreneurial activities in the crypto-arena. The main idea underlying this approach is that blockchain can create digital information units with elements of a property right. These new decentralized assets are also referred to as blockchain crypto properties (BCPs), which can be transferred via a protocol (MME 2018); cryptocurrencies can be viewed as BCPs.

The evolution of ICOs and the associated regulatory and economic implications are of theoretical, practical, and policy interest. BCPs are considered novel and groundbreaking in many ways; from a practical legal point of view, BCPs possess many physical and tangible properties (e.g., store of value); however, BCPs also have purely digital characteristics (e.g., zero distribution costs and algorithm-based). BCPs are a new and revolutionary concept and can be considered a new way of thinking about money and transactions. BCPs also have unique characteristics from fundraising and investment points of view. Unlike initial public offerings (IPOs), most ICOs are not currently regulated or audited. In some cases, unidentified individuals issue ICOs. While IPOs entail shares in operating companies, ICOs issue tokens for projects that have not yet been developed (Adham 2017).

ICOs also differ significantly from ECF. Crypto-token purchasers have a right to vote on future decisions related to a project (Dickson 2017); however, they do not own a part of the company. Blockchain’s decentralized nature allows an entrepreneur to reach directly to investors without ECF platforms like SeedInvest. In this way, blockchain helps create higher value by enabling an entrepreneur’s fundraising actions and changing the nature of their work.

Thus, there are sufficient differences between ICOs and other fundraising methods, such as IPOs or ECF. Due to blockchain’s disruptive nature and the high complexity of BCPs, policy responses to ICOs need to differ from other financing and investment mechanisms and tools, such as FDI, VC, ECF, and SWFs; however, regulations related to BCPs as assets or ICOs as a fundraising tool have received little theoretical or empirical attention. Thus, ICOs may provide a suitable setting to learn more about the relationship between entrepreneurship and institutions; however, the existing literature provides little information regarding policy responses to ICOs.

This paper offers important insights into the contexts and mechanisms associated with policy response to deal with disruptive technologies (Haveman et al. 2001). We explained how countries struggle to respond rapidly and benefit from disruptive technologies when forced to choose strategies within the constraints defined by the existing game rules. Such constraints do not apply to small countries with a homogeneous population (e.g., tax havens) that lack monitoring and sanctioning activities to ensure compliance with regulations.

Fourth, this study provides insights into the functioning of ICOs, which is especially important due to the early stage of research in the area and the lack of theoretical underpinning. In this regard, a parallel can be drawn with early research on VC. Drover et al. (2017) noted that most early VC research was highly descriptive. The articles focused on the VC process and the roles of key players. The concepts and frameworks developed in the early stage of this field’s development helped develop the foundations for subsequent research.

Fifth, prior research has suggested different mechanisms by which an entrepreneurial ecosystem can be developed (Spigel 2017). Some economies have chosen ICOs as one of the many ways to develop an entrepreneurial ecosystem. This article addresses some learning mechanisms essential to a nation’s ability to utilize ICOs for entrepreneurship development.

The article is organized as follows. We first discuss some background, concepts, and facts about blockchain and ICOs. The section following this provides a literature review. Next, we discuss the methods employed in the study. Then we explain the findings and develop some propositions, followed by a section on discussion and implications. In the final section, we offer conclusions.

## Blockchain and ICOs: some background, concepts, and facts

Table 1 provides definitions of key terms and concepts used in this article. As mentioned, to raise money through ICOs, a startup creates cryptocurrency or crypto-tokens utilizing blockchain, a decentralized ledger. After a block of records is entered into the ledger, the block’s information is mathematically connected to other blocks; in this way, a chain of immutable records is formed (Yaga et al. 2018). Due to this mathematical relationship, a block’s information cannot be changed without changing all blocks in the chain. Altering the information in a block would create a discrepancy likely to be noticed immediately by others in the network. Only authorized users with access to the information identities can be verified using cryptography-based signatures. Transactions are signed with “private keys” and “public keys,” created using complicated algorithms; thus, blockchain-based ledgers do not require record-keepers to trust each other.

**Table 1:** Explanation of major terms used in the paper

Term	Explanation
Blockchain	A decentralized ledger that maintains digital records of a transaction simultaneously on multiple computers
Crypto-token	A unit of value issued by a project or company, which rewards token owners. It allows the owner to perform particular actions (e.g., get a specific service on the network)
ERC-20 token	A technical standard used for smart contracts. It keeps track of token owners. It can be created with less than 100 lines of codes (Wolfson 2017)
Ethereum	A public blockchain-based open software platform, in which each node can be discovered by and known to other nodes in the network. It has its own cryptocurrency: Ether
ICO	A fundraising tool that allows a company to pre-sell future cryptocoins in exchange for cryptocurrencies of immediate and liquid value (e.g., bitcoin and Ether). A start-up raising money through ICOs can create its own cryptocurrency utilizing blockchain protocols. Roadmap goals and strategies are outlined in a whitepaper. ICO values are set up based on the amount of money required to achieve the stated objectives. The pre-sold tokens could serve as the medium of exchange in the future on a peer-to-peer platform (Li and William 2018)
Smart contracts	A “computerized protocol that executes the terms of a contract” (Szabo 1994)

A crypto-token denotes a unit of value issued by a project or a company, which rewards the token’s owner. For instance, crypto-tokens can be used to get a specific service on the network.

Smart contracts and ICOs are closely related because such contracts facilitate the minting, distribution, sale, resale, and use of tokens. Smart contracts execute automatically when certain conditions are met. In general, implementing smart contracts is among blockchain’s most transformative applications. A smart contract contains the logic that defines ICOs’ rules, such as how cryptocurrencies are collected and stored (e.g., until the investment goal is reached), the point at which it can complete the airdrop (distributes free tokens to eligible recipients), and how the issued tokens are to be used for services that the ICO is planning to offer.

While smart contracts utilized by many ICOs run on Ethereum (Fenu et al. 2018), the first blockchain to implement such contracts, Bitcoin is considered the first accounting ledger to be shared globally (MIT Technology Review 2017). While Bitcoin stores data related to transactions, Ethereum stores diverse types of data, such as those related to finance, industry, legal, personal information, community, health, education, and governance. In Ethereum, computers (nodes) connected in an open and distributed network verify and record transactions, providing the processing power needed to run smart contracts. Smart contracts are “installed” in each node, which allows users to interact with other nodes. The data can be accessed and used by computer programs known as decentralized applications (dApps), which is a significant difference between apps and applications hosted by a centralized organization. In the latter, while the codes may be distributed across multiple servers, a single entity controls them. For instance, while Facebook is a centralized app controlled by Meta Platform, a central entity does not control dApps.

The codes are generally open source, meaning anybody can use them to create a new dApp choosing their own ‘rules’ for ownership, transaction formats, and other aspects that may underlie the interactions among various parties. For example, Ethereum can be customized to offer unique solutions to particular needs. Some Ethereum-based successful dApps include Golem, Augur, and Melonport. Ethereum can be viewed as the first shared global computer.

Ethereum-based Ethereum Request for Comments 20 (ERC-20) is a technical standard used for smart contracts (Fenu et al. 2018), which keeps track of token owners at a given time (Consensys Media 2017). It defines a set of functions implemented by ERC20 compatible tokens to integrate them with other smart contracts or wallets; an ERC20 token can be created with less than 100 lines of code (Wolfson 2017).

## Literature review

Researchers have started to explore nonmarket environments (Baron 1995; Engelen et al. 2015; Miller and Friesen 1983; Porter 1990, 1996), mainly formal institutions related to ICOs. Prior research has noted that ICOs pose risks due partly to the lack of precise regulatory mechanisms in most jurisdictions (Chohan 2017; Conley 2017; Kaal 2018). Due to the nascent regulations, most ICOs “rely on legislative loopholes or, more accurately, what the issuing entity hopes (or prays) is a loophole or grey area” (Zetzsche et al. 2018, p. 11).

Unsurprisingly, researchers have noted pervasive fraudulent practices in ICOs (Hornuf et al. 2021). For instance, a study found that 40% of all ICOs destroyed investor value on the first day of trading (Momtaz 2020).

It is in the interest of most ICOs to register with regulatory agencies. For some firms, an attractive response would be to register in a tax haven jurisdiction (Marian 2019) to operate confidently without the fear of being investigated by regulators. Thus, tax havens allow intermediaries, such as cryptocurrency exchange platforms (e.g., Coinbase), to operate away from regulators by offering an “unregulated or lightly regulated environment” (Marian 2019, p. 16).

Research has just begun considering this new fundraising mechanism concerning formal institutions. Therefore, the current ICO research cannot help us understand the nature and sources of international variation in ICO-related formal institutions. The rest of this section describes formal institutions in the context of broader issues.

### Formal institutions and entrepreneurship development

Elements of the nonmarket environment, specifically formal institutions (such as minimal rules, tax incentives, availability of training and counseling services, government programs to enhance skills and education, technological services, and other administrative measures), influence the success of startups (Keuschnigg and Nielsen 2001).

Financial and fiscal incentives, such as lower taxes, play critical roles in attracting FDI (Blomström et al. 2003). The reduction ofReducing “hassle costs,” such as those associated with corruption and administrative inefficiency, can also stimulate FDI (Mallampally and Sauvant 1999).

Attracting funding, especially from specialized financial agencies like venture capitalists with the potential to generate high yields (Keuschnigg and Nielsen 2003), has been a key policy issue. Prior research has suggested that countries with lower capital gains taxation can attract more early stage and high-tech VC investments (Da Rin et al. 2006). The EU has been trying to stimulate the VC market through tax incentives and other measures targeted at the supply and demand sides (Cumming et al. 2017).

An issue relevant in this paper’s context is how formal institutions evolve in response to disruptive technologies, which are viewed as exogenous shocks (Haveman et al. 2001). In such cases, there are no recommended policy guidelines or templates to follow to increase national entrepreneurial activity (Greenwood and Hinings 1993).

### Formal institutions and the selection process of entrepreneurial ventures

Institutions affect the quality of entrepreneurial activities. In the EU, economies, subsidies, and other programs primarily target innovative industries (Keuschnigg and Nielsen 2003). While subsidy programs exist in many countries to encourage startups, they do not necessarily stimulate entrepreneurial activities.

Selective research and development (R&D) subsidies, based on systems committed to competitive principles provided to new technology-based firms, can have substantially positive economic impacts (Grilli and Murtinu 2014, 2018). Some jurisdictions have developed principles, guidelines, and criteria for selecting firms to receive subsidies. To evaluate applications for subsidies, the Agency for Innovation by Science and Technology in Flanders (IWT-Flanders), a governmental agency established by the Flemish Government in Belgium’s Flemish Region, has developed several criteria. Subsidies cover some costs of startups. The support rate was 50%, with a maximum subsidy of 250,000 Euros. The criteria include innovation, knowledge acquisition, quality of execution, commercialization potential, the value added to the Flanders region, and the firm’s financial viability (Meuleman and Maeseneire, 2012).

A society’s power structure and the vested interests of powerful actors affect how illegal and destructive entrepreneurial activities are defined and policed (Brownstein 2000). Regulators often decide whether certain funds should be allowed; for instance, prior research has suggested that authoritarian regimes are against decentralized fundraising, such as CF (Kshetri 2015).

Institutions often determine the type of entrepreneurial activities that may flourish. Some institutions are more likely to promote productive entrepreneurial activities, while others encourage destructive entrepreneurship (Baumol 1990; Stenholm et al. 2013). Some policymakers have been concerned with some categories of foreign investments potentially linked to unproductive and destructive entrepreneurial activities**.** Such investments include SWFs, which are state-owned investment funds invested in real and financial assets (e.g., stocks, bonds, real estate, or precious metals), or alternative investments such as private equity funds or hedge funds (Johan et al. 2013). Two major concerns have been expressed regarding SWFs: opacity and politicization (Murtinu and Scalera 2016). Regarding the first concern, skeptics suggested that, due to confidentiality practices, SWFs from some economies may be hiding some “threatening secrets” (El–Kharouf et al. 2010). Second, some critics argue that SWFs may have hidden political agendas (Murtinu and Scalera 2016); thus, SWFs in strategic sectors or critical infrastructures can pose national security threats (Drezner 2008).

Some SWFs are associated with adverse political, financial, and economic consequences (Drezner 2008). In 2006, Norway’s Government Pension Fund, managed by Norges Bank Investment Management, shorted the stocks of Iceland’s banks. This negatively affected Iceland’s economy (Setser 2008). Likewise, SWF investment was a key trigger that led to the 2006 coup in Thailand (Drezner 2008). Singapore’s SWF Temasek invested 1.9 billion USD in Shin Corp, owned by Thailand’s Prime Minister Thaksin Shinawatra’s family. Shin Corp allegedly paid no taxes on profits (Burton 2006).

Others are less concerned about the potentially destructive effect of SWFs. Some states are willing to allow SWFs or other funds that may promote low-quality or destructive entrepreneurship. In response to some countries’ opposition to SWFs, managers of such funds stated that they could easily invest elsewhere, as some countries badly need them (Drezner 2008). A similar pattern has been noted in ICOs; for instance, An et al. (2019) found no relationship between the rule of law score and the amount of capital raised by ICOs.

### Inter-jurisdictional competition

States tend to engage in regulatory competition (Konisky 2007), especially to attract investments from MNEs (Foss et al. 2019). Generally, corruption negatively affects entrepreneurship (Dutta and Sobel 2016; Liu et al. 2018); however, the exact relationship between corruption and entrepreneurship is unclear and depends on other contextual factors (Uribe-Toril et al. 2019). For instance, Mohamadi et al. (2017) found that government efficiency moderates the relationship between corruption and entrepreneurship development. In this paper’s context, even countries with high levels of corruption may benefit from blockchain-related entrepreneurship if they introduce new policy measures to attract crypto-ventures more efficiently than other economies. The mobile nature of assets and other resources allows MNEs to shift activities across jurisdictions (Foss et al. 2019). Firms can engage in regulatory arbitrage and move capital, human resources, and technology to the country with the most favorable regulations (Vogel 1996).

As mentioned, newly emerging funding mechanisms such as CF and VC, which focus on early stage companies, including some innovative ventures, can generate significant spillover externalities commonly associated with innovations (Agrawal et al. 2014; Keuschnigg and Nielsen 2003). Attracting such investments has been a key policy priority for many governments worldwide. For instance, European policymakers realized that such financing mechanisms increase the birth and growth of high-tech firms, which are critical to raising living standards, revitalizing the economy, and catching up with international competitors in innovation capabilities (Grilli and Murtinu 2014, 2015).

#### Tax havens and regulatory competitions

From the standpoint of regulatory competition for investments, tax havens, which offer a minimal tax liability for foreign individuals and businesses, possess unique characteristics. Many tax havens have been attractive destinations for FDI (Jones and Temouri 2016), while their extremely low-tax rates result in unfair competition with other jurisdictions. Some researchers have suggested that such jurisdictions are parasitic and cause a decline in the revenues of other countries (Slemrod and Wilson 2009).

Second, corporate tax cuts may attract the wrong kinds of investments, mainly motivated by profit-shifting, but may not result in tangible benefits to the broader economy (Shaxson 2016). In most tax havens, only a small segment of the population may benefit from foreign investments; there is often a lack of clear positive benefits to the national economy. In some tax havens, high-salaried finance jobs attract skills and talent; thus, they have had detrimental effects on other economic sectors, such as tourism (Christensen et al. 2016). Tax havens’ attempts to attract foreign investments may also degrade the entrepreneurial climate. For instance, according to Zucman (2015), Luxembourg’s role as a leading tax haven has benefited foreign expats at the expense of locals.

### Trade and industry associations’ roles

Nascent industries lack well-developed regulatory agencies (Powell 1993). In such cases, industry bodies and trade associations may fill the regulatory vacuum (Kshetri 2015). Their participation in the national policymaking arena is critical for the success of the industries they represent (Kshetri and Dholakia 2009).

Various mechanisms and factors are involved in the roles of industry bodies and trade associations in filling the regulatory vacuum and strengthening formal institutions. One role of trade associations is to monitor their members’ compliance with normative and coercive expectations (Greenwood et al. 2002). In emerging economies, trade associations also replace consultancy firms’ roles in filling the institutional voids (Back et al. 2014). They may also lobby to convince policymakers to introduce legislative measures to facilitate the growth of new areas, such as crowdfunding (Kshetri 2015). In some situations, the nation-state also finds it necessary to collaborate with professional associations to “rationalize” an arena of activity (Scott 1992, p. 211).

Trade associations can also play the role of institutional entrepreneurs by acting as institutional change agents (Kshetri and Dholakia 2009). Theorization, defined as “the development and specification of abstract categories and the elaboration of chains of cause and effect,” is an essential mechanism through which institutional entrepreneurs bring changes (Greenwood et al. 2002, p. 60). Two key elements of theorization are framing and justifying. Framing focuses on the need for change and justifies the proposed changes’ value for concerned actors (Greenwood et al. 2002). Overall, trade associations can be a vital force in changing ICO policies.

## Methods

Relatively little research involves ICOs. In areas like this, much initial research needs to be qualitative and concept- and theory-building (Eisenhardt 1989). This study utilizes inductive analysis (Thomas 2006), aiming to summarize raw data and capture key themes, processes, and patterns. The product of the inductive process consists of a model of the ICO phenomenon intended to help organize a further investigation of its components and holistic behaviors.

### Raw data

This study’s data mainly involved articles, blogs (from popular media), reports (e.g., the Swiss blockchain law firm MME’s report on BCPs), and policy documents from several governments. Archival data are among various recognized data sources for academic research (Ansari et al. 2016; Eisenhardt and Graebner 2007).

We took several precautions to ensure data quality. As recommended by prior researchers*,* we analyzed the sources of evidence and the evidence (Gottschalk 1969; Kshetri 2018c). We started with the “10 Must Read Bitcoin and Blockchain Blogs and Webpages” of Fintechnews Switzerland (FTNS 2016, Table 2) (http://fintechnews.ch/) (May 13, 2016). A search in Google Scholar indicated that FTNS had been widely cited in academic research.

**Table 2:** FTNS’s 10 must read bitcoin and blockchain blogs and webpages

	Source	Articles selected for analysis
1	CoinDesk	Hajdarbegovic (2014), Hertig (2017), Higgins (2016, 2017a, b, c), Matonis (2014), Milano (2018), O'Leary, 2017a, b), Reutzel, (2018); Rizzo (2014), Simpson (2017), Stanley (2017a, b, c), Sundararajan (2017). Tian (2017a, b), Zhao (2018a, b)
2	Bitcoin Magazine	bitcoinmagazine.com (2018), Marinoff (2018)
3	The LTB Network (https://letstalkbitcoin.com/)	letstalkbitcoin.com (2014)
4	Brave New Coin	Galka (2018), Lielacher (2018), Parker (2016, 2017)
5	CryptoCoinsNews (CCN)	Das (2017)
6	NewsBTC	newsbtc.com (2016a, 2016b, 2017), Yashu (2017)
7	AVC	Wilson (2017)
8	Ripple Insights	Zagone (2017)
9	MoneyBeat (The Wall Street Journal)	Vigna (2014)
10	FT Alphaville (The Financial Times)	Atkins (2018), Scaggs (2018), Waters (2017)

The FTNS’s must-read sources mainly include new outlets focusing on cryptocurrencies and blockchain and established news media, such as the *Wall Street Journal* (WSJ) and *Financial Times* (Table 2). While these outlets do not specifically focus on ICOs and cryptocurrency-related regulations, we found a sufficiently high number of articles covering regulatory aspects of ICOs in various jurisdictions.

Table 3 includes some main criteria that Gottschalk (1969) suggested for evaluating the evidence and its sources. The sources chosen in this paper are respected by peers; for instance, WSJ’s MoneyBeat (Vigna 2014) has cited news from CoinDesk (The #1 source in FTNS). Articles published by newsbtc and others cited Brave New Coin, while Ripple Insights has been covered by newsbtc and other established sources.

**Table 3:** Evaluation of data quality. *Source* Gottschalk (1969), Joselyn (1977), Kshetri (2018c)

Criterion	Explanation	Example
Time elapsed between events and reporting	Most newspaper articles were published the same day or the next day of the key policy-related event (e.g., the new legislation signed into law, the new policy approved, etc.)	A statement released on November 14, 2017 by the MAS regarding circumstances under which crypto-tokens could be considered to be securities according to Securities and Futures Act (SFA) and the Financial Advisers Act was published the next day (Sundararajan 2017)
Openness to corrections	Corrections are incorporated in many outlets we used	Washingtonpost’s corrections are stated after: “Correction to this article”
Range of knowledge and expertise of the person reporting the events	We used articles written by knowledgeable reporters/journalists	We cited one article by coindesk.com reporter Sundararajan (2017) but she wrote hundreds of articles about blockchain/cryptocurrency The sources and authors respected by their peers: WSJ’s MoneyBeat (Vigna 2014) cited news from Coindesk, newsbtc and others cited Brave New Coin, Ripple Insights has been covered by newsbtc and other established sources
Corroboration from multiple sources	Data and information were triangulated from multiple sources. We also visited the original source as suggested by Joselyn (1977)	Original sources allowed to make updates and corrections: Zagone (2017) reported that Mark Carney was the Chair of the FSB but updated information on the FSB website (https://www.fsb.org/profile/mark-carney/) stated that that was no longer the case

We utilized each news website’s search function to look for relevant articles. To locate articles related to ICO regulations, we searched using various combinations of keywords such as “ICO,” “cryptocurrency,” “blockchain,” “policy,” “regulations,” and “laws.” The minimum requirement for inclusion was at least one ICO- or cryptocurrency-related action taken by a regulatory agency. CoinDesk had the most articles, but we found at least one article in each of the other sources. The analyzed articles covered June 2014 to May 2018; we chose this period because the first ICO was launched in 2013, and regulators needed time to think about and adjust to the changes. Ethereum raised over 18 million USD in 2014, the largest ICO until that time (bitcoinmagazine.com 2018).

Like in snowball sampling, when we read an article recommended by FTNS, we followed links to other articles. Additional sources found this way, such as businesstimes.com.sg, bakermckenzie.com, wired.co.uk, lexology.com, and fastcompany.com, have also been cited in academic research.

We repeated the process until we developed a coherent set of themes representing regulatory and policy actions on the crypto front. After eliminating the redundant items, we analyzed 68 items, mainly popular press articles*.* The relevant materials from various sources resulted in 152 pages of text. They also included video recordings of interviews with people with experience in ICOs (about 23 min). The sources used in the analysis are marked with an asterisk (*) in the reference list.

Gottschalk (1969) suggested corroborating information from multiple sources; thus, data and information were triangulated from many sources. One of the best ways is to go to the source **(**Joselyn 1977). Therefore, whenever possible, we verified the information from the websites of the relevant regulatory agencies, such as the Monetary Authority of Singapore (MAS) (MAS 2017a, b), the UK’s Financial Stability Board (FSB), the US SEC (SEC 2017), The Swiss Federal Council (Werder 2017), the Government of Mauritius (Government of Mauritius 2019) the South Korean financial regulator, and the Financial Services Commission (FSC).^1^ The sources also allowed us to make updates and corrections, as emphasized by Gottschalk (1969). For instance, visiting the FSB website (https://www.fsb.org/profile/mark-carney/), we found that Mark Carney was no longer the FSB Chair, as reported in Zagone (2017).

As Joselyn (1977) emphasized, we assessed the possibility of bias. For instance, we excluded an article from a source recommended by FTNS due to a bias in the information a Western blockchain company provided regarding its plan to collaborate with a country’s government. We visited the country’s official websites, which had a lot of blockchain-related plans and activities; however, the sites contained no information related to the collaboration.

### Data analysis

Three broad tasks have been suggested for data analysis: data reduction, data display, and drawing conclusions (Miles and Huberman 1994). Regarding the first task, researchers must describe procedures to create meaning in complex raw data (Thomas 2006). They can do so by developing summary themes or categories. In the process of data reduction, categories that emerge from the coding of raw data have five features: a) category label (a word or a phrase to refer to the category); b) category description (meaning of the category, key characteristics, scope, and limitations); c) texts associated with the category (examples that illustrate meanings, associations, and perspectives), d) links (a category’s relation with other categories); e) the model embedding the category (Thomas 2006).

**Fig. 1:**
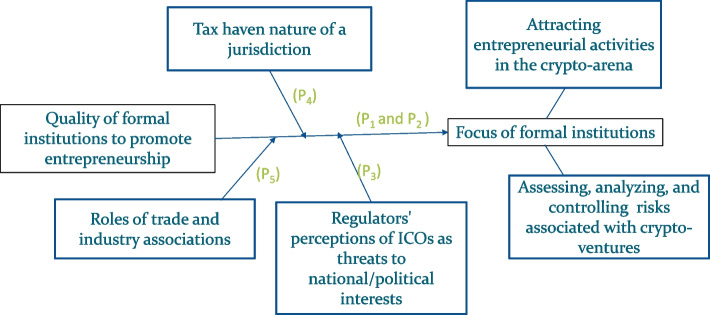
International variation in formal institutions related to initial coin offerings

Figure 1 presents the category labels, represented by boxes. We followed the coding process that Thomas (2006) suggested and read and reread the text to generate key categories. Due to a low amount of data, the process was manageable without software, and the data were manually coded.

As suggested by prior researchers, we revised and refined the category system (Kshetri 2018c). Under a given category, we also searched for subtopics. In addition, conceptual themes were searched within the sub- topics to provide new insights. Moreover, as Thomas (2006, p. 242) suggested, we combined categories with similar meanings under a “superordinate” category. For example, formal institutions are “superordinate” categories with several categories (Fig. 1).

### Writing the findings

Prior researchers suggested including detailed descriptions of the categories that emerged from the data and appropriate quotations to illustrate the categories’ meanings (Kshetri 2018c; Thomas 2006). Tables 4 and 5 explain the categories’ meanings and characteristics.

**Table 4:** Key categories related to non-market environment emerged from data analysis

Category	Meaning/explanation	Implications in relation to institutional theory (“rules of the game” (North 1990), and **“**rule setting, monitoring and sanctioning activities” (Scott 1995: 42))
Tax havens	Jurisdictions that offer minimal *tax* liabilities for foreign individuals and businesses. They often have politically and economically stable environments. They share little or no financial information with other jurisdictions	Compared to Western economies, they provide more favorable rules of the game for illegal and illicit activities
Quality of institutions to promote entrepreneurship	“Regulatory efficiency and quality” required to “enhance business activity” (WB, 2019)	The rules of the game favor productive entrepreneurship
Perceived threat to national/political interests	Policy makers’ tendency to regard and interpret some aspects of ICOs as a potential cause of economic and/or social damage	In order to minimize such threats, they are likely to monitor and evaluate the actions of crypto-ventures and develop appropriate sanctioning systems
Crypto-related trade association	An organization founded by businesses that operate in the crypto industry	The goal is to develop rules of the game that favor the crypto industry

**Table 5:** Key categories related to institutional response emerged from data analysis

Category	Sub-category (Meaning/explanation)	Examples	Implications in relation to institutional theory
Formal institutions to promote entrepreneurial activities in the crypto-arena (Laws, regulations, policies and administrative provisions are geared toward stimulating ICOs)	Fiscal incentives and administrative efficiency (*lowering tax rates* and regulatory burdens for starting and operating a business)	Low tax rates in Switzerland’s Zug Canton, Singapore, Gibraltar and other jurisdictions Switzerland’s plan to allow small fintech firms to conduct business without seeking authorization (Werder 2017)	Crypto-ventures can exploit specific opportunities within the existing rules of the game. In addition, regulators have devised mechanisms which potentially redefine the rules of the gam**e** in order to attract crypto-ventures
	Legal clarity of crypto-tokens (degree of clarification of legal position regarding different types of crypto-token and certainty of their legal protection)	Switzerland’s FINMA a has identified three categories of ICOs and clarified which will be subject to securities law requirements Singapore’s MAS provided several case studies of security and non-security crypto-tokens with illustrations Bermuda’s plan to enact bespoke laws for ICOs	These regulators are clarifying the rules of the game in order to reflect the legitimacy of crypto-tokens and streamline enforcement
	Measures to enrich blockchain ecosystem (complex relationships of blockchain firms with key players such as government agencies, industry and trade association, consumers, investors, financial institutions and capital markets and other ingredients)	Canton of Zug: investment in education and efficient infrastructure. Blockchain startups provided with a competitive hiring environment	For disruptive technologies, the rules of the game may need to be changed to include the government's proactive roles in the development of essential ingredients. While negative sanctioning (punishment) focuses on undesired effects, positive sanctioning (reward) can stimulate the growth of this industry
Formal institutions to assess, analyze, and control risks associated with crypto-ventures (Laws, regulations, policies and administrative provisions are geared toward minimizing ICOs’ costs to the national economy)	Regulatory monitoring of ICO ventures (The existence of a system to observe and check the quality of crypto-ventures to ensure that they do not violate existing regulations)	The U.S., Bill H.R. 4752: aims to establish an independent Financial Technology Task Force to examine whether and how cryptocurrencies would finance terrorism and subsequently propose regulations to counter such activities	Regulators are creating and establishing new rules of the game to ensure that ICOs and cryptocurrencies are not used to harm the national interest They want to put in place mechanisms to monitor and evaluate the actions of crypto-ventures and develop a negative sanctioning system that punishes the violators
	Sandbox approaches (A government program for crypto-ventures to test new services and products with *partnership and supervisory* roles of regulators)	The U.K’s FCA and Canada’s CSA launched “sandbox” programs for blockchain projects	Regulators are looking at how new rules of the game can be introduced in order to encourage the growth of legitimate crypto-ventures and avoiding and neutralizing potential negative consequences

Figure 1 presents the outcome as a model incorporating essential categories. The constructs and relationships in Fig. 1 are based on insights from the data. Together with the testable propositions, they represent a theory regarding the relationships among key ICO concepts.

### The nature of theory developed

A theory is a “statement of relations among concepts within a boundary set of assumptions and constraints” (Bacharach 1989, p. 496). This paper’s theory is what Gregor (2006) refers to as Type IV theory, which explains and predicts a phenomenon. This paper explained constructs related to dependent and independent variables, their associations, and the states covered. This approach can help researchers and practitioners develop a more refined understanding of ICO-related policies and enable reliable and accurate prediction. Future empirical testing may support or refute the theory presented in this paper.

Prior researchers suggested that the boundary conditions related to a theory’s assumptions should be specified (Bacharach 1989; Whetten 2002). Whetten (2002) suggests two types of assumptions: conceptual and contextual. Conceptual assumptions are the “implicit whys underlying an explicit answer to a specific why question” (p. 58). This paper’s foundational institutional theory assumes that institutional actors seek legitimacy from various groups and “accept and follow social norms” (Tolbert and Zucker 1996, p. 176).

Conversely, contextual assumptions determine the conditions that circumscribe the explanation proposed by the theory and hence specify a theory’s boundary (Whetten 2002). A theory covers only a particular class or state of things (Weber 2012). A strong theory can “discern conditions in which the major proposition or hypothesis is most and least likely to hold” (Sutton and Staw 1995, p. 376). In other words, contextual assumptions explain when, where, and for whom a given theory holds (Whetten 2002). The contextual boundary of this paper’s model comprises ICOs, the processes of raising and investing in ICOs, and the institutions in which the fundraisers and investors operate. The context does not include other types of fundraising activities. One additional point is that the proposed associations among the various constructs in Fig. 1 are expected to be positive or negative on a ceteris paribus basis.

## Findings and propositions

This section is organized according to Fig. 1.

### Quality of entrepreneurship-related institutions

By quality of entrepreneurship-related institutions, we mean “regulatory efficiency and quality” required to “enhance business activity” (WB 2019). Such institutions are a key component of the nonmarket environment (Baron 1995; Engelen et al. 2015; Miller and Friesen 1983; Porter 1990, 1996), affecting crypto-ventures’ location decisions. This section discusses the regulatory responses to ICOs in economies with high- and low-quality entrepreneurship-related institutions. Table 6 compares such responses of four jurisdictions: two with low- and two with high-quality levels of entrepreneurship-related institutions. Specifically, we use the Global Entrepreneurship and Development Index (GEDI)—which measures and ranks nations’ entrepreneurship climates (Acs et al. 2016)—and the World Bank’s Doing Business ranks (WB 2019) as proxies to assess the quality of such institutions.

**Table 6:** Comparing institutional responses of jurisdictions with different quality levels of entrepreneurship-related institutions

Economy	Indicators related to entrepreneurship related institutions	Some activities on the ICO front	Implications in relation to institutional theory (“rules of the game” (North 1990), and “rule setting, monitoring and sanctioning activities” (Scott 1995: 42))
The U.S	WBDB 2017 rank: 8 GEDI score (rank): 85.0 (1)	July 2017: SEC concluded that some coins were structured as securities and thus they were required to register with the agency, which forced some crypto-ventures to close down	The regulators performed monitoring roles to ensure that crypto-ventures do not operate in ways that undermine the existing rules of the game
South Korea	WBDB 2017 rank: 5 GEDI score (rank): 50.5 (27)	September 2017: FSC announced a plan to ban all forms of virtual currencies and ICOs (Kim, 2017) noting that they are overly speculative and constitute a “violation of the capital market law” (O'Leary 2017b)	
Puerto Rico	WBDB 2017 rank: 55 GEDI score (rank): 48.9 (51)	Issued a license for a Cryptocurrency International Financial Entities Government officials have especially emphasized the openness of the Island’s economy to blockchain and cryptocurrency industry Citing unfavorable laws in the U.S., many U.S.-based crypto-entrepreneurs moved to Puerto Rico	Lack formal controls such as sanctioning and monitoring systems to control and minimize illegal/illicit activities: more favorable rules of the game for such activities compared to the U.S. and Korea
Panama	WBDB 2017 rank: 70 GEDI score (rank): 32.2 (118)	The lack of regulative clarity around token sales but some major ICOs have been launched Crypto-ventures registered in Panama can operate without fear of government coercion and regulatory enforcement. A company registered in Panama was Havelock Investments dubbed as Bitcoin “stockmarket” or “proto-ICO” 2014: many U.S.-based crypto-ventures were fined by the SEC and forced to close down for marketing securities and offering shares without registering with the commission. Panama-registered companies such as Havelock engaged in similar activities but operated with impunity	

#### High-quality entrepreneurship-related institutions

Regarding what institutional theorists describe (North 1990; Scott 1995), the rules of the game and sanctioning activities in countries with high-quality entrepreneurship-related institutions ensure that (minority) investors are protected (LaPorta et al. 2002). Thus, crypto-ventures should be subject to the same game rules; however, this is a challenging task. ICOs differ significantly from other fundraising methods, such as IPO and ECF. Crypto-token purchasers have a right to vote on future decisions related to a project (Dickson 2017); however, unlike in an IPO or an ECF, they do not own a part of the company. Thus, crypto-token holders cannot often control and influence the directors’ actions.^2^ As noted above, ensuring crypto-venture regulatory compliance is even more important due to a high degree of fraud proneness in ICOs (Hornuf et al. 2021) and because many ICOs destroy investor value on the first day of trading (Momtaz, 2020). All of these underscore the importance of regulators in monitoring ICOs. For instance, US regulators closely monitor ICO activities (Table 6). In mid-2018, national regulators in 40 jurisdictions, including many US states and Canadian provinces, launched a probe dubbed “Operation Cryptosweep.” They cracked down on fraudulent ICOs, opened about 70 investigations, and warned 35 companies about violating securities laws (Kshetri 2018a). The SEC has been active in investigating and controlling fraudulent ICOs. The July 2017 SEC report directly responded to the attack on the decentralized autonomous organization (DAO) hub, determining that DAO tokens were securities (Shin 2017). In October 2017, the SEC announced that it would prosecute the creator of two ICOs—REcoin and DRC—that were allegedly stock-like structures (Morris 2017). The main goal of this ruling was to protect smaller investors from overextending themselves in the ICO market and investing in fraudulent projects (Galka 2018).

Regulators have sanctioned and monitored activities (Scott 1995) to ensure that crypto-ventures comply with the existing rules of the game. The ex-CEO at Coinapult noted that rules related to money service businesses and money transmitter businesses force foreign companies to block US investors (letstalkbitcoin.com 2014). Compliance requires enormous investments and burdensome activities. Laws may not support lax monitoring, lenient sanctions, and non-compliance in promoting crypto-ventures.

Monitoring and sanctioning activities to enforce the game’s rules are even more apparent in economies like South Korea (Table 6), which have banned ICOs outright. While such activities put excessive, one-sided emphasis on controlling risks and essentially ignore the importance of innovations (Deng et al. 2018; Zetzsche et al. 2018), these extreme measures have been justified to ensure adherence to the rules of the game.

These governments believe regulations should be established after carefully evaluating ICOs’ cost-benefit; some countries have adopted regulatory sandbox approaches to address this. Within the sandbox, startups test new services and products under the supervision of regulators (Higgins 2016). The goals are often to facilitate product testing and promote consumer safety to minimize destructive consequences.

In 2014, the UK Financial Conduct Authority announced several blockchain and cryptocurrency projects to its regulatory sandbox. Likewise, in 2017, Canadian Securities Administrators launched a new FinTech “sandbox” program, aiming to encourage blockchain and other FinTech firms (Higgins 2017a). The goal was to “curate” an environment where companies could test new kinds of blockchain-based financial products without affecting the broader marketplace (Higgins 2017). In the words of institutionalists (North 1990; Scott 1995), these monitoring activities aim to ensure that crypto-ventures play by the existing game rules.

To attract blockchain innovators, in 2016, Mauritius began establishing a regulatory sandbox license (RSL) (Stanley 2017a). The RSL “offers the possibility for an investor to conduct a business activity for which there exists no legal framework, or adequate provisions under existing legislation in Mauritius.” Based on the above discussion, the following proposition is presented:

*P*_*1*_ In jurisdictions with high-quality entrepreneurship-related institutions, formal institutions’ primary focus is more likely to be on assessing, analyzing, and controlling risks associated with crypto-ventures compared to jurisdictions with low-quality entrepreneurship-related institutions.

#### Low-quality entrepreneurship-related institutions

Some policymakers are concerned with foreign investments such as SWFs (Johan et al. 2013); however, countries with less developed entrepreneurial ecosystems need to tackle more severe challenges than those arising from such investments. As noted above, despite the overall negative effect of corruption on entrepreneurship (Dutta and Sobel 2016; Liu et al. 2018; Uribe-Toril et al. 2019), even corrupt governments can help develop entrepreneurship if they efficiently introduce new policy measures (Mohamadi et al. 2017). For instance, Puerto Rico (Table 6) has shown a high interest in attracting crypto-entrepreneurs and investors following Hurricane Maria; the government wants to diversify its economy (Reutzel 2018). Such economies are likely to adopt game rules (North 1990) that are favorable to new firms. Puerto Rico’s attractive tax incentives include zero federal personal income taxes, zero capital gains tax, and low business taxes (Bowles 2018).

Such economies are more likely to set up new game rules (North 1990) that incentivize activities, such as crypto-ventures. Regarding sanctioning and monitoring (Scott 1995) of new ventures, they are likely to be less concerned about potential negative consequences. For instance, Puerto Rico desperately needs FDI and has seen an opportunity in blockchain technology. The Island’s government officials have emphasized the openness of the Island’s economy to the blockchain and cryptocurrency industries (Reutzel 2018).

The state closely monitors crypto-ventures’ actions in economies with high-quality entrepreneurship-related institutions; however, monitoring and sanctioning activities are lacking in economies with low-quality entrepreneurship-related institutions. Regarding the state’s monitoring activities (Scott 1995), crypto firms are subject to lax monitoring in the latter groups.

The game rules (North 1990) often result from bargaining between politicians and entrepreneurial firms (Dagher 2018). Blockchain companies likely enjoy higher bargaining power vis-a-vis the governments in economies with low-quality entrepreneurship-related institutions. In other words, the propensity to compete to attract investments (Konisky 2007; Vogel 1996) is likely higher in countries with low-quality entrepreneurship-related institutions (Fig. 1). Panama (Table 6) has attracted many crypto-ventures despite lacking specific regulations concerning ICOs (FTNS 2018). While the US and many other jurisdictions have been cracking down and getting tough on cryptocurrencies and blockchain, several crypto-ventures have launched ICOs in Panama (FTNS 2018; Yashu 2017), including Decent.bet (online sports betting and casino), Monster Byte (cryptocurrency gaming platform), Prime-Ex Perpetual (real estate tokens) and Orocrypt (precious metals tokens). These observations support An et al. (2019), who found no relationship between the rule of law score and the amount of capital raised by ICOs in an economy. Crypto-ventures are attracted to economies that belong to the upper box on the right side of Fig. 1, despite the low rule of law scores because such economies provide incentives to such ventures. Additionally, crypto-ventures are also attracted in economies that belong to the lower box on the right side because of the high rule of law scores, characterized by better entrepreneurial ecosystems.

Generally, countries with low-quality entrepreneurship-related institutions lack what institutional theorists (North 1990; Scott 1995) refer to as formal controls, such as sanctioning and monitoring systems to minimize illegal and illicit activities. Thus:

*P*_*2*_ In jurisdictions with low-quality entrepreneurship-related institutions, formal institutions’ main focus is more likely to be on promoting entrepreneurial activities in the crypto-arena than those with high-quality entrepreneurship-related institutions.

### Regulators’ perceptions of ICOs as threats to national/political interests

Some sources of financing, such as SWFs, are perceived to serve the countries’ political interests and hidden agendas (El-Kharouf et al. 2010; Murtinu and Scalera 2016; Drezner 2008) and thus are viewed as political and economic threats. A point worth noting is that following the September 11, 2001 attacks*,* the US intensified measures against terrorist financing (Biersteker and Eckert 2007; Zetsche et al. 2018). Some regulators consider that ICOs exhibit economic and political concerns of similar magnitude. Critical features of cryptocurrencies, such as anonymity and irreversibility of transactions, may facilitate terrorist acts by funding entities owned or controlled by terrorists (Brill and Keene 2014).

Like some types of SWFs (Drezner 2008), ICOs may be associated with possible political, financial, and economic consequences. In contrast, some analysts have feared that SWFs might possess “threatening secrets” (El–Kharouf et al. 2010); furthermore, ICOs’ risks, such as terrorist financing and money laundering, have been of concern. States are unsurprisingly exercising their power by enacting new rules and measures, enforcing existing rules, and monitoring and sanctioning activities (Groenewegen and Van der Steen 2007; Scott 1995) to minimize threats to national and political interests.

In the US, the (H.R. 4752) “Financial Technology Innovation and Defense Act” bill was introduced into Congress in 2018 to establish an independent Financial Technology Task Force. The task force’s charge would be to examine whether and how cryptocurrencies would finance terrorism and propose regulations to counter such activities (Zhao 2018a, b, c).

Monitoring is a crucial feature of regulative institutions (Scott 1995). Economies like China have channeled resources and discourse to implement additional monitoring levels to ensure that crypto-ventures do not threaten political and national interests. The need for heightened monitoring is justified since many Chinese citizens use cryptocurrencies to circumvent strict capital controls due to the depreciating Yuan (newsbtc.com 2016). In 2017, officials from China’s central bank, the People’s Bank of China (PBoC), reportedly visited the offices of the country’s largest crypto exchanges to identify whether the exchanges were satisfying the anti-money laundering (AML) and capital control requirements (Zhao 2018b).

Moreover, the rules of the game (North 1990) in authoritarian regimes are against decentralized funding, such as CF (Kshetri 2015). In addition to decentralization, cryptocurrencies’ anonymity and privacy may increase political concerns. Thus, ICOs thus may face higher risks of more severe sanctioning and additional monitoring. This is because the Chinese Communist Party (CCP) is concerned that ICOs could threaten traditional power players by changing the nature of the CCP’s control over the population and its interactions with firms. For instance, ICOs may allow firms to overcome regulatory obstacles and access VC-type funding in new ways. Thus, the CCP views cryptocurrencies as a means to subvert state power (Tian 2017a).

ICO-funded firms may build projects or protocols to compete with incumbent businesses, or they may provide censorship-resistant alternatives. All these mean that state control may erode (Hackett 2017). The PBoC argued that many ICOs were “covers” for illicit activity (Tian 2017b); as a result, about 85 ICOs were shut down in 2017 (Marinoff 2018).

Most ICOs use legislative loopholes (Zetzsche et al. 2018). Some governments may take legislative or enforcement actions to close such loopholes if these actions contradict other institutional policies and expectations. Seo and Creed (2002, p. 226) call this phenomenon “intra-institutional conformity that creates inter-institutional incompatibilities.” Here is how it may operate. Creative and innovative mindsets that see the value in ICOs, called cognitive institutions, and policy measures to promote entrepreneurship, which are regulative institutions (Scott 1995), are internally compatible; however, they are incompatible with other game rules, such as international money laundering and terrorist financing laws and political hostility toward decentralized fundraising systems. The above leads to the following:

*P*_*3*_ The perceived threat to national/political interests has a positive moderating effect on the relationship between the quality of institutions and the focus on assessing, analyzing, and controlling risks associated with crypto-ventures.

### Tax haven jurisdictions

The effect of the quality of entrepreneurship-related institutions on attracting entrepreneurial ventures in the crypto-arena is especially apparent in tax haven jurisdictions. Regarding the characteristics of institutions, there is criticism that they allow tax havens to act parasitically, causing a decline in other countries’ revenues (Slemrod and Wilson 2009). While such criticism may be valid, the rules of the game in tax havens provide low-regulation and low-tax jurisdiction for new ventures, which some founders of blockchain projects prefer (Marian 2019). For instance, to operate in Gibraltar, crypto firms must pay application fees of 12,500 to 37,500 USD; the same fee must be paid annually along with other supplementary fees (gfsc.gi 2017). Many tax haven jurisdictions are tiny and thus are well-suited to serve entrepreneurial activities that do not require complex digital and physical infrastructure. Blockchain applications are virtual and operate via nodes distributed worldwide (Marian 2019). While the US and some other jurisdictions, as noted above, have sanctioning and monitoring systems (Scott 1995) to track potentially illegal activities, many tax haven jurisdictions lack such formal control systems.

The market, as well as nonmarket components of the environment, affect a firm’s strategy (Baron 1995; Engelen et al. 2015; Miller and Friesen 1983; Porter 1990, 1996); conversely, due to ICOs’ virtual nature, the firm’s location is largely irrelevant from the market perspective. Nonmarket factors, such as low tax and the lack of sanctioning and monitoring systems, make tax havens attractive destinations for locating ICO activities.

Due to factors like social homogeneity, such economies also exhibit a higher degree of responsiveness to change and flexibility compared to bigger economies (Read 2001). Their ability to rapidly redefine the rules of the game (North 1990; Scott 1995) allows them to create a resource-rich institutional environment in which the government takes legislative, regulatory, administrative, and fiscal measures to facilitate the availability of key ingredients needed for firms (Feldman and Kelley 2002; Keuschnigg and Nielsen 2001). An example is Switzerland, especially its Zug Canton. In 2017, the Swiss Federal Council initiated a move to amend the country’s Banking Act and Banking Ordinance to reduce market entry barriers for FinTech companies and strengthen their competitiveness. The plan would allow small FinTech firms (blockchain-based and others) that accept up to 1 million Swiss franc (CHF) (1.02 million USD) from customers to conduct business without seeking authorization (Werder 2017).

Prior researchers have established significant relationships between tax incentives, such as lower capital gains taxation, and the level of early stage and high-tech VC investments (Da Rin et al. 2006; Cumming et al. 2017). Many popular ICO destinations offer favorable tax treatment to ICOs. For instance, Zug Canton, where the Crypto Valley is located, has a tax rate of 14.6%, among the country’s lowest (gibraltarlaw.com 2018). Switzerland is touted as a cryptocurrency haven. Four of the world’s ten largest ICOs were located in Switzerland in 2017 (Atkins 2018). Companies that have launched ICOs in Singapore pay 17% corporate tax, and there is no capital gains tax (RESSOS 2018). Likewise, Gibraltar’s corporate tax is 10% (gibraltarlaw.com 2018).

A further benefit of tax havens is that their small size and homogeneity (Read 2001) allow them to respond quickly and develop ecosystems needed for new areas of economic activities. These include government initiatives, such as programs to enhance skills and education, technological services, and legislative, regulatory, and administrative measures (Keuschnigg and Nielsen 2001). Zug is well known for its heavy investment in education and efficient infrastructure (Chadwick 2018). The competitive hiring environment attracted more than 200 FinTech startups, primarily based on blockchain, as of November 2018 (ambcrypto.com 2018). For instance, MME needed to assemble a team of experts in diverse areas, such as technology, banking, corporate law, tax, and AML, to develop its proposal on BCP (Müller et al. 2017).

Administrative measures to facilitate the success of startups are a key component of formal institutions (Keuschnigg and Nielsen 2001; North 1990). In 2016, Zug started accepting cryptocurrency payments for public services (newsbtc.com 2016a). Such measures facilitate the ease of operations of crypto-ventures. Likewise, the Mauritius government has collaborated with the private sector in the country and international companies to develop a blockchain ecosystem (Government of Mauritius 2019). Key focus areas included KYC (know your client) rules, digital identity, and title registries. In the subsequent phase, it plans to build a talent pool of developers, entrepreneurs, executives, and regulators to enrich the ecosystem (Stanley 2017b; newsbtc.com 2017).

Their size, social homogeneity, and flexibility (Read 2001) have also allowed tax havens to rapidly introduce new rules of the game (North 1990; Scott 1995), which are especially important for new activities like ICOs. For example, some tax havens provide legal clarity to crypto-tokens, which has helped ICO promotors provide effective signals to attract investors. For instance, the Swiss financial watchdog, Financial Market Supervisory Authority (FINMA) has), identified three ICO categories of ICOs: (a) Payment ICOs (function as means of payment, need to comply with AML regulations); (b) Utility ICOs (provide access rights to applications or service); (c) Asset ICOs (function in the same manners as equities/bonds). Under Swiss laws, (a) and (b) would not be treated like financial securities, but (c) is subject to securities law requirements if they satisfy certain conditions (e.g., paying dividends/interest or giving claims to earnings streams) (Atkins 2018).

In 2017, the MAS published a statement explaining various crypto-token models (MAS 2017a, b). The report also), which contained several case studies with illustrations. One example of a non-security crypto-token was tied to a computing power-sharing platform—another crypto-token was connected to a startup investment fund that counted as a security (Sundararajan 2017).

Bermuda expressed a desire to be “one of the first countries…to specifically regulate ICOs” (Milano 2018). Likewise, according to the Gibraltar Financial Services Commission, Gibraltar was among the first jurisdictions to have bespoke crypto-token rules (gfsc.gi 2017).

Research shows that states engage in regulatory competition (Konisky 2007). Such an approach is especially apparent in tax havens, where providing legal clarity regarding crypto-tokens has become a key competition area.

An important question is how policymakers in tax haven jurisdictions view and deal with what is referred to as destructive entrepreneurship (Baumol 1990) associated with BCPs. The state’s power (Groenewegen and Van der Steen 2007) is exercised to minimize the potentially negative effects of ICOs on the local economy. In 2017, Puerto Rico issued a banking license for Cryptocurrency International Financial Entities, which prohibits such entities from conducting business with persons or businesses in Puerto Rico and is one of the territory’s most powerful international banking and financial services structures (Reeves 2017). Such entities can offer international banking, brokerage, investment management, and financial services from Puerto Rico to clients outside the territory. Puerto Rican policymakers know that the new policies may not necessarily attract actors that engage in high-quality entrepreneurial activities.

Overall, the game’s rules and sanctioning and monitoring activities (North 1990; Scott 1995) in tax havens are designed to attract new firms. Various contradictions (Seo and Creed 2002) that led to monitoring and sanctioning ICOs in economies like China and the US do not exist in these economies. The existing and newly created rules of the games have provided certainty regarding BCPs, low regulations, and minimal or no monitoring of crypto-related activities, which have been attractive for blockchain-based firms. Thus, we propose:

*P*_*4*_ The tax haven nature of jurisdiction has a positive moderating effect on the relationship between the quality of institutions and the focus on promoting entrepreneurial activities in the crypto-arena.

### The presence of trade and industry associations

National legal systems related to crypto-entrepreneurship are underdeveloped. In nascent areas, trade and industry associations, considered to be a key element of the nonmarket environment (Baron 1995; Porter 1990, 1996), introduce voluntary instruments and ethical principles, such as codes of conduct and other mechanisms to influence regulations (Kshetri and Dholakia 2009). For instance, trade associations lobby to convince regulators to introduce legislative measures to facilitate the growth of the industry they represent (Kshetri 2015, 2018c).

In ECF, influencing regulations has been a significant goal of the National Crowdfunding Association of India, the African Crowdfunding Association, and the Danish crowdfunding Association (Kshetri 2018c). The National Crowdfunding Association of the US played a key role in enacting the JOBS (Jumpstart Our Business Startups) Act (Kshetri 2015). Similar developments have occurred in the crypto-arena. Switzerland’s Crypto Valley Association (CVA) is an obvious example of blockchain-related trade associations. The CVA has engaged local government, startups, VC investors, and other key actors, initiated research projects, and organized conferences, hackathons, and other industry events (Parker 2017).

The Russian government announced a plan to form a similar trade association: the Russian Association of Blockchain and Cryptocurrency (RABIK). The RABIK would reportedly work with regulators to develop policy and increase the “legitimization” of the technology (O’Leary 2017a; b). Through framing and justification (Greenwood et al. 2002), these associations can present their ideas that connect regulations with positive economic results and persuade policymakers of the importance of regulations (Kshetri and Dholakia 2009). The RABIK, for instance, argued that the Russian economy lost 310 million USD in the first ten months of 2018 due to a lack of ICO regulations (https://news.bitcoin.com/russian-economy-18-billion-rubles-ico-regulation/). Such activities can influence the regulators to redefine the game’s rules that favor crypto-ventures.

High-performing and exemplary organizations are also likely to frame a need for a change and justify it to make the game’s rules favorable to the industry (Kshetri and Dholakia 2009). One such example is the Swiss blockchain law firm MME, a member of the CVA, which released the “Conceptual Framework for a Legal and Risk Assessment of BCP” in 2017 (Müller et al. 2017). The report’s main objective is to assess and analyze crypto-venture risks. MME uses a functionality-based method to assess BCPs’ legal and tax implications and evaluate associated risks and investment suitability. MME argues that its method can be considered in all jurisdictions, irrespective of legal and regulatory frameworks. The CVA distributed the MME report on BCP, explaining where the Swiss law stands in each BCP type. Such actions give crypto-ventures an accurate understanding of regulatory systems for BCPs, helping promote entrepreneurial activities in the crypto-arena (Fig. 1).

An association’s high-performing and exemplary members can act as institutional entrepreneurs (Kshetri and Dholakia 2009). MME’s report on BCP is helping to develop a shared understanding of various kinds of crypto-tokens among regulators to enact enforceable legislation, which can help assess, analyze, and control risks associated with crypto-ventures (Fig. 1). The analysis can help policymakers take measures to promote crypto-entrepreneurship and provide standard tools and techniques for token issuers and investors to evaluate and communicate risks. The expert power (Kshetri and Dholakia 2009) is effectively channeled to change the rule of law in their favor.

Sometimes, an industry group, trade associations, and regulators try to achieve the same goals (Kshetri and Dholakia 2009). The FINMA in Switzerland has emphasized the importance of protecting investors (swissinfo.ch 2018). The CVA’s codes of conduct also aim to achieve this, emphasizing the importance of codes of conduct to foster best practices and fight scams (Simpson 2017*).* It has provided guidelines that new ICOs must follow to reduce unethical practices (Jones 2018). For example, the International Standards Organization (ISO) has formed Technical Committee 307 (ISO/TC 307) to work in the areas of blockchain and distributed ledger technologies. One of the focus areas of ISO/TC 307 has been identity management, and some important standards are expected to be developed soon, which can address various regulatory concerns (Hersey 2022).

An optimal game rule can be explicated by combining the state’s coercive power (Groenewegen and Van der Steen 2007) and the expert power of trade associations and industry bodies (Kshetri and Dholakia 2009). Thus, it is proposed that:

*P*_*5*_ The presence of trade and industry associations has a positive moderating effect on the relationship between the quality of institutions and the focus on promoting entrepreneurial activities in the crypto-arena.

## Discussion and implications

Prior research emphasized the importance of formal and informal institutions in shaping entrepreneurial financing tools and investment instruments, such as ECF (Kshetri 2015, 2018c), FDI (Blomström et al. 2003; Mallampally and Sauvant 1999), VC (Cumming et al. 2017; Keuschnigg and Nielsen 2003; Da Rin et al. 2006), and SWFs (Johan et al. 2013; Murtinu and Scalera 2016; El–Kharouf et al. 2010; Drezner 2008). Conversely, the above discussion suggests that different mechanisms are involved in institutions’ effect on ICOs. Due to the disruptiveness of blockchain, the possibility of contradictory social and economic effects complicates ICOs, leading to a wide range of policy preferences across multiple countries.

Regarding institutions (North 1990; Scott 1995), some regulators are redefining the game’s rules and penalizing crypto-ventures through enacting laws and measures hostile to ICOs. Some regulators have performed monitoring roles to ensure that crypto-ventures do not operate in ways that undermine the existing rules of the game. They have realized that additional monitoring is needed to minimize harming the national economy.

While the state is a powerful institutional actor (Groenewegen and Van der Steen 2007), international pressures facing small economies indicate that the state’s power is limited. By imposing international sanctions, major world economies have challenged the game’s rules in tax havens; however, blockchain may allow tax havens to circumvent international sanctions.

Cryptocurrencies can arguably act as tax haven alternatives (Marian 2013). The central idea here is that potential tax evaders obtain similar advantages from cryptocurrencies that tax havens offer; cryptocurrencies’ decentralized feature means that no central authority can impose a tax, and high levels of anonymity and privacy mean that users do not need to identify themselves (Kshetri 2018b). That is, users are relatively free from government monitoring. Compared to tax havens, cryptocurrencies are less vulnerable to pressure from developed countries’ regulators (Marian 2013). While more negligible risks are involved in buying and selling cryptocurrencies without registering in a jurisdiction, doing so has higher risks in fundraising activities, such as ICOs. The nature of the rules of laws and sanctioning and monitoring activities (North 1990; Scott 1995) in tax havens offer attractive destinations for ICOs.

The above discussion indicates that other entrepreneurial policies may complement or substitute ICOs. The effect of policies to develop a rich blockchain ecosystem may be complementary because crypto-ventures see increased opportunities to enter such markets. Mechanisms adopted by tax havens, such as financial secrecy and the lack of corporate transparency and policy infrastructures developed to support such activities, may also act as complementary mechanisms. In contrast, policies to stimulate the VC market through tax incentives and other measures, such as those in the EU (Cumming et al. 2017), may act as a substitute for ICOs. Policymakers may prefer to focus on VCs that attract innovative ventures rather than investments of unproven quality, such as those associated with ICOs.

Next, we investigate our research questions. Regarding RQ1, mitigating potentially harmful economic and social impacts has become a major focus of ICO policy discussions in some economies. Regarding regulatory responses, ICOs have strong similarities and striking differences with other investment and financing models, such as SWFs and CFs. Some underlying concerns are similar to what researchers have found in SWFs (Murtinu and Scalera 2016; El-Kharouf et al. 2010), such as cryptocurrencies being used to harm political interests or national security.

Still, SWFs and ICOs differ concerning the nature of their potential threats. Whereas critics have argued that foreign governments may undermine a nation’s political, economic, and financial stability through SWFs, facilitation of terrorism financing has been a concern with ICOs. Other concerns, like in CF (Kshetri 2015; Mollick 2014), include those related to investor protection. Due to these concerns, nations have been slow to enact ICO-related regulations. Some nations have chosen an outright ban on ICOs to address these concerns. Pilarowski and Yue (2017) discussed the reasons behind China’s ban on ICOs, which included the difficulty faced by the government in monitoring the cryptocurrency market due to the anonymous nature of cryptocurrencies, evasion of foreign exchange controls, and potential instability in the financial system from manipulation in the prices of cryptocurrencies. Furthermore, as with CF (Kshetri 2015), policymakers in countries characterized by strong social, political, and economic controls are against ICOs due to their decentralized nature, high levels of anonymity, and privacy.

Some governments have seen tremendous opportunities in ICOs and have created ICO-friendly environments through tax policies favoring crypto-ventures and clear regulations to attract such ventures. Such policies have attracted a larger number of crypto-entrepreneurs.

Concerning RQ2, the international policy divergence can be attributed partly to differences in nations’ economic and institutional characteristics, which lead to different weights of benefits and costs associated with ICOs. Like other funding mechanisms, such as CF and VC (Agrawal et al. 2014; Keuschnigg and Nielsen 2003), ICOs can generate positive spillover externalities; however, inappropriate uses and activities associated with ICOs can also lead to negative externalities. These negative externalities are likely to be viewed less negatively by regulators in countries with lower-quality entrepreneurship-related institutions. Moreover, in the case of tax havens like Puerto Rico, such negative externalities often do not affect investors in the host countries because the firm launching an ICO engages in little or no business activities in the tax haven.

Countries with higher-quality entrepreneurship-related institutions are likely to have developed principles, guidelines, and criteria for providing government support and incentives for entrepreneurial firms (Grilli and Murtinu 2014; Meuleman and Maeseneire 2012). These criteria often attract and reward efficient and competitive firms. These countries have been reluctant to jump into ICOs too quickly because some illicit entities may use nefarious fundraising tactics to victimize unsuspecting investors. Worse, some governments are concerned that ICOs may work against national and political interests due to blockchain’s decentralization and anonymity. In some cases, the interests and values of powerful actors and their different interests and contrasting methods of understanding have led to diverse policies.

Countries with lower-quality entrepreneurship-related institutions are less likely to support productive entrepreneurial activities (Baumol 1990; Stenholm et al. 2013). Some such countries are determined to benefit from the opportunity that ICOs can provide. For instance, Puerto Rico has seen blockchain as a great window of opportunity to create a diversified economy and attract foreign investments. Unsurprisingly, tax haven economies have generally been more optimistic about the benefits of ICOs and less concerned about the potential risks.

While stringent policy and enforcement measures exist in more conventional financing and investment mechanisms, such as FDI (Blomström et al. 2003; Mallampally and Sauvant 1999), VC (Cumming et al. 2017; Keuschnigg and Nielsen 2003; Da Rin et al. 2006), ECF (Kshetri 2015), and SWFs (Johan et al. 2013; Murtinu and Scalera 2016; El-Kharouf et al. 2010; Drezner 2008), ICOs are characterized by nascent regulative institutions. Startups raising money through ICOs are incentivized to engage in opportunistic behavior, especially in jurisdictions with weak regulatory and enforcement environments. For instance, the SEC has maintained that some crypto-tokens could be considered securities required to comply with the disclosure requirements. Many other jurisdictions lack such requirements. As a result, nefarious firms, unfortunately, may prefer to operate in weak regulatory and enforcement environments.

Like in other economic sectors (Kshetri and Dholakia 2009), trade associations, such as the CVA, play a key role in developing ICO-related formal institutions. Trade associations can identify mechanisms by which ICOs can benefit the economy by conducting research, organizing conferences, and other events. They can work closely with government agencies and other actors and help them understand such benefits. Like the roles played by CF-related trade associations in Africa, Denmark, India, and the US (Kshetri 2015, 2018c), ICO-related trade associations may engage in lobbying activities to convince policymakers to introduce legislative measures to facilitate ICOs. Indeed, these associations can be more prominent in shaping the ICO market due to the newness.

### Managerial implications

Formal institutions related to ICOs have implications for financial innovation and international management. Recent research has focused on the role of contextual factors as a determinant of an ICO’s success (Chitsazan et al. 2022). Among relevant contextual factors are legal and regulatory frameworks and local government sentiments (Chitsazan et al. 2022). Startups and established firms find it attractive to register and launch ICOs in jurisdictions with predictable and clear regulations, such as the legal clarity of BCPs. In such jurisdictions, the initiators of ICO projects can include the key legal provisions in a white paper, providing legal guarantees to investors (Kasatkin 2022).

Furthermore, jurisdictions that take administrative measures (Feldman and Kelley 2002; Keuschnigg and Nielsen 2001) to facilitate the availability of key ingredients needed for blockchain firms are preferable from the perspective of locating higher-end activities, such as headquarters and R&D facilities, and management employees. In such jurisdictions**,** retaining, attracting, and hiring key personnel such as blockchain lawyers, code writers, and researchers can be easy. Blockchain firms should also examine other positive sanctioning mechanisms that can stimulate the growth of this industry (e.g., Switzerland’s policies allow small blockchain-based FinTech firms to conduct business without seeking authorization) (Werder 2017).

Other critical elements of the nonmarket environments (Baron 1995; Miller and Friesen 1983; Porter 1990, 1996)), such as active industry bodies and trade associations, can help develop formal institutions related to ICOs. By instituting industry codes of conduct, trade associations, such as the CVA, can encourage best practices and fight scams and thus work as a substitute for formal regulative institutions. Fighting scams is especially critical because fraudulent practices have been reported in many ICO projects (Florysiak and Schandlbauer 2022; Swartz 2022). These should also be considered key factors in shaping blockchain firms’ location decisions.

Startups launching an ICO should remember that nonmarket factors, such as clear regulatory protections and well-developed ethical codes from trade associations, may serve as a quality signal of the ICO as an investment option. This is important since the newness and complexity involved in blockchain and cryptocurrencies make it difficult for investors to interpret the signals. The idea is to send positive signals and avoid engaging in actions that could cause others to make unfavorable judgments (Ang and Brau 2003). ICO promotors can follow securities markets and IPOs; for instance, corporate insiders hide or delay disclosing unfavorable information to sell securities at higher prices (Megginson and Weiss 1991). An example is the issuance of secondary shares offered by pre-IPO owners such as investors and employees. Due to potentially harmful information conveyed by secondary shares, some insiders under-file such shares in the original filing. Amendment filings may be submitted in future data, which are less noticeable (Ang and Brau 2003). If opportunistic insiders think the demand for shares would be high, they may submit an amended filing in which secondary shares increase and primary shares reduce, or both secondary and primary shares increase, but the former accounts for most of the increase (Ang and Brau 2003). Switzerland’s FINMA has published guidelines that ICOs must adhere to, and ICOs are regulated under AML laws or as securities. In this way, ICO jurisdiction can contribute to an attractive value proposition for investors by assuring their investments are legally protected.

Understanding ICO-related regulations worldwide is vital to deciding to whom the tokens can be marketed and sold. For instance, German jurisdiction is likely based on whether the ICO is marketed in Germany, e.g., ICO information is in German, on a German website, or distributed to potential investors in Germany (Sigle 2017). ICOs launched in foreign countries may not be able to sell tokens to US investors. Various rules force foreign companies to block US investors (letstalkbitcoin.com 2014).

### Policy implications

Like other funding mechanisms, such as CF and VC (Agrawal et al. 2014; Grilli and Murtinu 2014, 2015), the hope is that positive effects can be achieved through ICOs. As researchers have found in other settings (Gale and Luo 2004; Lin and Khattak 2021), with appropriate policy interventions, governments can encourage entrepreneurial activity in the crypto-arena and eliminate the national security, political, and economic risks. Appropriate policy support mechanisms are needed to attract entrepreneurial activities in the crypto-arena. To enrich the entrepreneurial ecosystem around ICOs, governments can combine investment subsidies and loans for blockchain and crypto startups. Governments should also collaborate with universities and other academic institutions to develop a blockchain and crypto workforce.

Strong enforcement measures are needed to reduce national security, political, and economic risks. Training law enforcement agencies to investigate and prosecute crypto crimes must be a priority. Governments can collaborate with the private sector to develop criminal justice and legal actors, such as lawyers, judges, and prosecutors.

Policymakers should also undertake initiatives to create awareness of crypto fraud; educated consumers are less likely to fall victim to crypto fraud. Such measures are likely to achieve the same or better effects than sanctioning and monitoring activities, raising the likelihood of catching offenders.

### Future research directions

This paper focused on several factors that may affect the ICO trajectory, such as an economy’s entrepreneurial performance, perceived threat to national/political interests, and tax haven nature; however, we did not evaluate the effects of other key factors that might further explain the evolution ICO regulatory trajectory. This research did not examine the contexts, mechanisms, and processes of ICO-related policymaking, responses to and impact of ICOs and ICO policies, and cross-state competition collaboration and learning. In this section, we identify some critical areas for future research.

#### Characteristics of a nation

This article discussed how small tax havens are taking several initiatives to encourage ICOs. The issues to be considered in future research include ICO-related regulatory responses of small economies. Prior research suggests that formal institutions such as competition policy in small economies must be tailored and designed to suit their markets (Gal 2003). Due to factors like social homogeneity, such economies also exhibit a higher degree of responsiveness to change and flexibility than bigger economies (Read 2001). In an ICO context, ConsenSys founder Joseph Lubin said that compared to larger jurisdictions, smaller nations such as Mauritius have tools and “nimbleness” that are needed to rapidly adapt and react to changes required for new technologies (Stanley 2017a). Thus, future research can examine how the size of an economy could affect the nature of ICO-related regulatory responses.

This study focused on formal institutions in the context of ICOs. Scholars must expand the research lens to include informal institutions in future research. Prior research has suggested that informal institutions are as important as formal institutions in shaping economic activities, such as ICOs. In the context of this paper, Li et al. (2021) have noted that finance-related issues are affected by social and cultural norms. Entrepreneurs evaluate formal and informal institutions before engaging in specific entrepreneurial activities (Aidis et al., 2008). For instance, a challenge in China is that commercial organizations, such as those offering ICOs, are less trusted (Kshetri 2017). related point is that formal and informal institutions affect each other (Axelrod 1997). Some areas that researchers might pursue include how legislations affect how entrepreneurs view ICOs and how potential investors develop favorable or unfavorable attitudes toward ICOs.

Future researchers should also explore differences in various countries’ ICO-related institutions according to their historical context. For instance, due to regulatory asymmetry or jurisdictional arbitrage, economies with liberal regulations related to the repatriation of capital and profits may create a favorable crypto-entrepreneurship environment. According to a Deloitte report, Switzerland’s liberal regulations—especially the lack of state control over the repatriation of capital and profits—attract foreign multinationals and enterprises. Due to numerous federal and regional incentives for new foreign investors, the country is often used as a location for international headquarters and trading companies (Parker 2017). The blockchain company Xapo created a dedicated page (https://xapo.com/resources/switzerland/), which points out the top ten reasons for FinTech startups to move to Switzerland. One of the main reasons is Switzerland’s historical independence and insulation from foreign influence. The path dependence approach argues that different events steer history in a particular direction, influencing the path a nation undertakes (North 1990). This approach can provide a suitable analytical method for studying this phenomenon.

#### Contexts, mechanisms, and processes of ICO-related policymaking

This research looked at the nature and sources of divergence in ICO-related regulations. Future researchers might examine more detailed contexts associated with such regulations and policies. For instance, organizing ICO policy approaches in different jurisdictions into different analysis grids and graphical representations could provide a valuable means to highlight policy targets (e.g., ICOs’ productive, unproductive, and destructive consequences). Furthermore, such an organization can identify actors responsible for policy actions (e.g., central banks such as China’s PBOC and other regulatory bodies such as the US Securities and Exchange Commission (SEC)) and define intervention targets. Some crypto-entrepreneurs may think that specific regulations have been established because ICOs are viewed as possible threats to authoritarian power due to their decentralized nature. Such entrepreneurs may respond differently from those who think that the regulations are aimed at controlling fake ICOs and scams.

Prior researchers have argued that politicians consider many factors in policy formulation, such as the policy’s effect on the achievement of political and ideological goals and advancement of moral values, cost efficiency, and probability of success(Volden et al. 2008). Investigating how policymakers in countries with different institutions may view the potential impacts of crypto-ventures on these parameters differently may be an interesting topic for future research.

Policymakers often rely on information from inside and outside the nation to examine or test a policy’s appropriateness. Inside the nation, policymakers may look at public preferences, interest groups’ goals, political actors’ aims, and lessons learned from previous policies (Volden et al. 2008). Prior researchers have shown that organizational capabilities develop mainly through learning mechanisms, such as trial and error and the selection and retention of past behaviors (Zollo and Winter 2002). Thus, the mechanisms and processes associated with learning in ICO-related policymaking would provide a promising avenue for future research.

#### Responses to and the impact of ICOs and ICO policies

Prior research in developing countries has suggested that revenues from investments, especially in natural resources like minerals and oil development, can corrupt local elites (Haufler 2004). There is not much information available regarding the impact on local economies of revenues collected from foreign blockchain and cryptocurrency firms. In this regard, one area that future researchers may wish to pursue is examining the similarities and differences of ICOs’ effects on the local economy compared to other sources of financing.

Likely, the propensity to relocate to a state with a more favorable regulatory climate may be related to attitudinal factors. Baumol (1990) noted that an individual might engage in productive, unproductive, or destructive entrepreneurship depending on the incentive structures provided by formal and informal institutions. A more likely and logical explanation is that different individuals are likely to engage in entrepreneurial activities under different incentive structures provided by the nonmarket environment (Baron 1995; Porter^1990^, ^1996^). Future research can consider how attitudinal factors could affect an entrepreneur’s decision to locate ICO activities in jurisdictions with different formal institutions that provide different incentive structures.

Prior researchers have suggested that individuals’ response to regulatory regimes results from their perceptions of the regime’s legitimacy and the associated regulators in question (Braithwaite et al. 1994). For instance, investors and entrepreneurs who think ICO policies reduce fraud may respond differently than those who think such policies strengthen authoritarian rulers and elites. Another intriguing avenue for future research would be examining how individuals’ perceptions of legitimacy and fairness of ICO-related regulations link with the perceptions of regulators’ motivation.

#### Cross-state competition, collaboration, and learning

Prior researchers have noted that globalization has affected different policy domains differently (Janicke and Jacob 2004). For instance, regarding environmental policy, countries and companies with trade relations with countries with strict regulations were reported to have stricter policies themselves (Foljanty-Jost 1997). Porter (1990) argued that a strict environmental policy could improve the competitiveness of a country’s firms and sectors. First, by adopting a strict environmental policy, a country might achieve a competitive advantage if the policy subsequently diffuses internationally. Firms that have developed technologies to meet strict environmental standards can export their technologies, and their competitive advantage may stem from learning effects or patent protection for their innovation (Porter 1990; Porter and van der Linde 1995). Future research can address the applicability of this logic in the current context.

Regarding the mechanisms and processes of learning, policymakers also learn from the experiences of other countries. For instance, they may look at policies in other countries that have been successful under similar circumstances (Volden et al. 2008**)**. Prior researchers have referred to the spread of policies from one government to another as “learning-based policy diffusion.” They have argued that it is essential to properly characterize and evaluate this process to understand the context, conditions, process, and consequences of such diffusion (Volden et al. 2008**)**. At the same time, some nations (e.g., Switzerland) are less likely to be influenced by other nations. Thus, learning mechanisms in the context of ICO policies should be addressed in future research.

Another future research area, especially in the context of tax haven economies, involves the effect of a country’s approach to diplomacy and measures to attract global crypto-ventures. Prior research has suggested that international diplomacy affects the private sector, and diplomacy efforts are implemented and geared toward changing the behavior of foreign investors (Haufler 2004). For example, Mauritius’ diplomacy in trade preferences is impressive, which may partly explain its different orientation to crypto-regulations compared to other tax havens. The country is known for effective political institutions, and its parliamentary system builds consensus by representing all groups. The country’s scores were high in several measures of institutional quality, such as political participation, the rule of law, and control of corruption (Frankel 2010). Nations with large diplomatic networks are more likely to listen to and respect other countries’ legitimate security concerns than nations that lack such networks*.* Such nations may face pressure from other countries to take initiatives to develop regulations and enforcement activities to control the destructive consequences of crypto-ventures worldwide. Nations that take efforts to strengthen international diplomacy and cooperation with other countries are likely to take measures to control the potentially harmful consequences of ICOs. It is also possible to empirically examine links between a country’s diplomatic network size and its ICO-related actions. For instance, Lowy Institute for International Policy’s Global Diplomacy Index ranks the diplomatic networks of 42 countries that are G20 or OECD members (https://globaldiplomacyindex.lowyinstitute.org/). Similar indices can be constructed for other economies.

#### The effects of Coronavirus disease (COVID)-19 on policy response related to ICOs

Prior researchers have noted that interest in digital solutions has increased during the Covid-19 pandemic (Khan et al. 2021d; Campino et al. 2022). Related to this study, Lee et al. (2022) noted that the increase in cryptocurrency prices during the COVID-19 pandemic revived the ICO market. More broadly, Hong and Yoon (2022) found that the structure and properties of the networks in cryptocurrency markets drastically changed during the pandemic. Fraudulent activities have also increased in crypto-related areas, such as decentralized finance (DeFi) applications (Wilson 2020). DeFi applications rely on trustless and transparent protocols of decentralized networks, such as blockchain, to create financial products without intermediaries. All these are likely to attract policymakers’ attention. Thus, future researchers should explore the impact of pandemics, such as COVID-19, on formal and informal institutions from the standpoint of ICOs.

## Concluding comments

Due primarily to newness, regulators have different perspectives and viewpoints on the economic, political, and societal costs and benefits of crypto-entrepreneurship. The process by which such costs and benefits are perceived and evaluated differs among countries with varying quality levels of entrepreneurship-related institutions because policymakers must align economic, political, and other goals. Different governments are motivated and driven by different combinations of such goals, impacting their orientations toward crypto-entrepreneurship. For instance, unlike tax haven jurisdictions, countries with high-quality entrepreneurship-related institutions are only interested in high-quality and high-impact entrepreneurship, not just the type of entrepreneurship.

The existing game rules, such as those related to international money laundering and terrorist financing laws and political hostility toward decentralized fundraising systems, would negatively affect ICOs. Such concerns are less prevalent in many tax havens; however, these jurisdictions are better off if they take measures to develop a rich entrepreneurial ecosystem around blockchain and cryptocurrency instead of just focusing on tax incentives intended to attract low-quality crypto firms.

Finally, combining the state’s coercive power and trade associations’ expert power would effectively bring ICOs’ benefits to the economy with minimum economic, social, and political costs. Government–industry collaboration is especially relevant in promoting ICOs and crypto-ventures due to the current regulatory vacuum in these areas.

## Data Availability

Not applicable.

## Abbreviations

AML: Anti-money laundering
BCP: Blockchain crypto property
CCP: Chinese Communist Party
CF: Crowdfunding
CHF: Swiss franc
COVID: Coronavirus disease
CVA: Crypto Valley Association
DAO: Decentralized autonomous organization
dApp: Decentralized application
DeFi: Decentralized finance
ECF: Equity crowdfunding
ERC-20: Ethereum Request for Comments 20
FDI: Foreign direct investment
FINMA: Financial Market Supervisory Authority
FSC: Financial Services Commission
FTNS: Fintechnews Switzerland
GEDI: Global Entrepreneurship and Development Index
ICO: Initial coin offering
ICT: Information and communication technology
IPO: Initial public offerings
ISO: International Standards Organization
JOBS: Jumpstart Our Business Startups
KYC: Know your client
MAS: Monetary Authority of Singapore
PBoC: People’s Bank of China
R&D: Research and development
RABIK: Russian Association of Blockchain and Cryptocurrency
RSL: Regulatory sandbox license
SEC: Securities and Exchange Commission
SWF: Sovereign wealth fund
USD: US dollars
VC: Venture capital
WSJ: Wall Street Journal

## Footnotes

1 The statement was in the Korean language: http://www.fsc.go.kr/info/ntc_news_view.jsp?bbsid=BBS0030&page=1&sch1=&sword=&r_url=&menu=7210100&no=32085. We relied on the summary provided in O'Leary (2017b)

2 I thank a reviewer for suggesting this.

## References

[CR1] Acs ZJ, Szerb L, Autio E (2016). Global entrepreneurship and development index 2015.

[CR2] Adham M (2017) Backing a new digital currency: initial coin offerings. https://www.forbes.com/sites/forbesfinancecouncil/2017/05/23/backing-a-new-digital-currency-initial-coin-offerings/#3c766d981d90

[CR3] Agrawal A, Catalini C, Goldfarb A (2014). Some simple economics of crowdfunding. Innov Policy Econ.

[CR4] Aidis R, Estrin S, Mickiewicz T. Institutions and entrepreneurship development in Russia: a comparative perspective. J Business Ventur 2008;23(6):656–672. 10.1016/j.jbusvent.2008.01.005.

[CR5] ambcrypto.com (2018) Does crypto valley move to Asia?. https://ambcrypto.com/does-crypto-valley-move-to-asia/. Accessed 15 Nov 2018

[CR6] An J, Duan T, Hou W, Xu X (2019). Initial coin offerings and entrepreneurial finance:&nbsp;the role of founders’ characteristics. J Altern Invest.

[CR7] de Andrés P, Arroyo D, Correia R, Rezola A (2022). Challenges of the market for initial coin offerings. Int Rev Financ Anal.

[CR8] Ang JS, Brau JC (2003). Concealing and confounding adverse signals: Insider wealth-maximizing behavior in the IPO process. J Financ Econ.

[CR9] Ansari S, Garud R, Kumaraswamy A (2016). The disruptor's dilemma: TiVo and the U.S. television ecosystem. Str Man J.

[CR10] Atkins R (2018) ‘Crypto nation’ Switzerland issues guidelines to support market. Accessed 16 Feb

[CR11] Axelrod R (1997). The complexity of cooperation.

[CR12] Bacharach SB (1989). Organizational theories: some criteria for evaluation. Acad Manag Rev.

[CR13] Back YK, Parboteeah P, Nam D (2014). Innovation in emerging markets: the role of management consulting firms. J Int Manag.

[CR14] Baron DP (1995). Integrated strategy: market and nonmarket components. Calif Manage Rev.

[CR15] Baumol WJ (1990). Entrepreneurship: productive, unproductive, and destructive. J Polit Econ.

[CR16] Biersteker TJ, Eckert SE (2007). Countering the financing of terrorism.

[CR17] bitcoinmagazine.com (2018) What is an ICO?. https://bitcoinmagazine.com/guides/what-ico

[CR18] Blomström M, Kokko A, Mucchielli JL, Herrmann H, Lipsey R (2003). The economics of foreign direct investment incentives. Foreign direct investment in the real and financial sector of industrial countries.

[CR19] Bowles N (2018) Making a crypto utopia in Puerto Rico, Feb 2. https://www.nytimes.com/2018/02/02/technology/cryptocurrency-puerto-rico.html

[CR20] Braithwaite V, Braithwaite J, Gibson D, Makkai T (1994). Regulatory styles, motivational postures and nursing home compliance. Law Policy.

[CR21] von Briel F, Davidsson P, Recker JC (2018). Digital technologies as external enablers of new venture creation in the IT hardware sector. Entrep Theory Pract.

[CR22] Brill A, Keene L (2014). Cryptocurrencies: the next generation of terrorist financing?. Def against Terror Rev.

[CR23] Brownstein HH (2000). The social production of crime statistics. Justice Res Policy.

[CR24] Burton J (2006) Singapore may see worst fallout from Thai coup, Sept 20. https://www.ft.com/content/dd2afa6a-48b3-11db-a996-0000779e2340

[CR25] Campino J, Brochado A, Rosa Á (2022). Initial coin offerings (ICOs): why do they succeed?. Financ Innov.

[CR26] Chadwick S (2018) Canton Zug, May 10. https://cryptovalley.swiss/consensus-2018-sponsor-canton-zug/

[CR27] Chitsazan H, Bagheri A, Tajeddin M (2022). Initial coin offerings (ICOs) success: conceptualization, theories and systematic analysis of empirical studies. Technol Forecast Soc Change.

[CR28] Chohan UW (2017) Initial coin offerings (ICOs): risks, regulation, and accountability discussion paper series: notes on the 21st Century. Doi: 10.2139/ssrn.3080098. https://ssrn.com/abstract=3080098

[CR29] Christensen J, Shaxson N, Wigan D (2016). The finance curse: Britain and the world economy. Br J Pol Int Rel.

[CR30] Conley JP (2017) Blockchain and the economics of crypto-tokens and initial coin offerings. Vanderbilt University Department of Economics Working Papers 17-00008

[CR31] Consensys Media (2017). Making token sales smart. https://media.consensys.net/making-token-sales-smart-28fe2011512f

[CR32] Cumming DJ, Grilli L, Murtinu S (2017). Governmental and independent venture capital investments in Europe: a firm-level performance analysis. J Corp Finance.

[CR33] Dagher J (2018) Regulatory cycles: revisiting the political economy of financial crises, IMF Working Paper, WP 18/8

[CR34] Das S (2017) Indian P2P Car Share Startup Drivezy Raises $10 Million, Accepts Bitcoin & Announces ICO, OCTOBER 6, https://www.ccn.com/indian-p2p-car-share-startup-drivezy-raises-10-million-accepts-bitcoin-announces-ico/

[CR35] Deng H, Huang H, Wu Q (2018). The regulation of initial coin offerings in China: problems, prognoses and prospects. Eur Bus Organ Law Rev.

[CR36] Dickson B (2017) Can you trust crypto-token crowdfunding? https://techcrunch.com/2017/02/12/can-you-trust-crypto-token-crowdfunding/. Accessed 07 Sept 2017

[CR37] Drezner DW (2008). Sovereign wealth funds and the (in) security of global. J Int Aff.

[CR38] Drover W, Busenitz L, Matusik S, Townsend D, Aaron A, Dushnitsky G (2017). Equity financing research: venture capital, corporate venture capital, angel investment, crowdfunding, and accelerators. J Manag.

[CR39] Dutta N, Sobel R (2016). Does corruption ever help entrepreneurship?. Small Bus Econ.

[CR40] Eisenhardt KM (1989). Building theories from case study research. Acad Manag Rev.

[CR41] Eisenhardt KM, Graebner ME (2007). Theory building from cases: opportunities and challenges. Acad Manag J.

[CR42] El-Kharouf F, Al-Qudsi S, Obeid S (2010). The Gulf cooperation council sovereign wealth funds: are they instruments for economic diversification or political tools?. Asian Econ Pap.

[CR43] Engelen A, Schmidt S, Buchsteiner M (2015). The simultaneous influence of national culture and market turbulence on entrepreneurial orientation: a nine-country study. J Int Manag.

[CR44] FTNS (2016) Top 10 must read bitcoin and blockchain blogs and online publications, Fintechnews Switzerland. http://fintechnews.ch/blockchain_bitcoin/10-must-read-10-bitcoin-and-blockchain-blogs-webpages/3940/. Accessed 08 Sept 2017

[CR45] FTNS (2018) Cryptocurrencies, ICOs see growth in tax haven Panama, May 9, Fintechnews Switzerland. http://fintechnews.ch/blockchain_bitcoin/cryptocurrencies-icos-see-growth-in-tax-haven-panama/18365/

[CR46] Feldman MP, Kelley MR (2002). how states augment the capabilities of technology-pioneering firms. Growth Chang.

[CR47] Fenu GLM, Marchesi M, Tonelli R (2018) The ICO phenomenon and its relationships with ethereum smart contract environment. CoRR https://arxiv.org/abs/1803.01394

[CR48] Florysiak D, Schandlbauer A (2022). Experts or charlatans? ICO analysts and white paper informativeness. J Bank Financ.

[CR49] Foljanty-Jost G, Weidner H (1997). Die Bedeutung Japans für die vergleichende Umweltpolitikforschung- vom Modell zum Auslaufmodell? In Umweltpolitik und Staatsversagen. Perspektiven und Grenzen der Umweltpolitikanalyse.

[CR50] Foss NJ, Mudambi R, Murtinu S (2019). Taxing the multinational enterprise: on the forced redesign of global value chains and other inefficiencies. J Int Bus Stud.

[CR51] Frankel J (2010) Mauritius: African success story.&nbsp;NBER Working Paper No. 16569 December 2010. Doi:10.3386/w16569

[CR52] Gal MS (2003). Competition policy for small market economies.

[CR53] Gale A, Luo J (2004). Factors affecting construction joint ventures in China. Int J Proj Manage.

[CR54] Galka M (2018) The 10 largest ICO fund raises: successes, controversies and lessons learned, 10 May. https://bravenewcoin.com/insights/the-10-largest-ico-fund-raises-successes-controversies-and-lessons-learned

[CR55] gfsc.gi (2017) Distributed ledger technology regulatory framework (DLT framework), Gibraltar Financial Services Commission. http://www.gfsc.gi/dlt

[CR56] gibraltarlaw.com (2018) Where is the best country to launch an ICO – Switzerland or Gibraltar?. http://www.gibraltarlaw.com/ico-switzerland-gibraltar/

[CR57] Gottschalk L (1969). Understanding history: a primer of historical method.

[CR58] Government of Mauritius (2019) Blockchain technology and its impact on digital transformation of mauritius, 07 Feb. http://www.govmu.org/English/News/Pages/Blockchain-Technology-and-its-impact-on-Digital-Transformation-of-Mauritius.aspx

[CR59] Greenwood R, Hinings CR (1993). Understanding strategic change: the contribution of archetypes. Acad Manag J.

[CR60] Greenwood R, Suddaby R, Hinings CR (2002). Theorizing change: the role of professional associations in the transformation of institutionalized fields. Acad Manag J.

[CR61] Gregor S (2006). The nature of theory in information systems. MIS Q.

[CR62] Grilli L, Murtinu S (2014). Government, venture capital and the growth of European high-tech entrepreneurial firms. Res Policy.

[CR63] Grilli L, Murtinu S (2015). New technology-based firms in Europe: market penetration, public venture capital, and timing of investment. Ind Corp Change.

[CR64] Grilli L, Murtinu S (2018). Selective subsidies, entrepreneurial founders' human capital, and access to R&D alliances. Res Policy.

[CR65] Groenewegen J, Van Der Steen M (2007). The evolutionary policy maker. J Econ Issues.

[CR66] Hackett R (2017) 7 reasons why China banned ICOs. http://fortune.com/2017/09/05/china-bitcoin-blockchain-ico-ban/. Accessed 15 Sept 2017

[CR67] Hajdarbegovic N (2014) UK financial regulator's new initiative encourages bitcoin innovation. https://www.coindesk.com/uk-financial-conduct-authority-fca-launches-bitcoin-initiative/

[CR68] Haufler V (2004). International diplomacy and the privatization of conflict prevention. Int Stud Perspect.

[CR69] Haveman HA, Russo MV, Meyer AD (2001). Organizational environments in flux: the impact of regulatory punctuations on organizational domains, CEO succession, and performance. Organ Sci.

[CR70] Hersey F (2022) Global state of SSI and how to build on it: EU report, Jan 24. https://www.biometricupdate.com/202201/global-state-of-ssi-and-how-to-build-on-it-eu-report

[CR71] Hertig A (2017) The Father of the ICO Is All About Identity Now, December 4. https://www.coindesk.com/markets/2017/12/04/the-father-of-the-ico-is-all-about-identity-now/

[CR72] Higgins S (2016) UK regulator adds 9 blockchain startups to fintech 'Sandbox'. https://www.coindesk.com/uk-regulator-9-blockchain-fintech-sandbox/

[CR73] Higgins S (2017a) Canada's securities watchdog seeks blockchain firms for startup 'Sandbox'. https://www.coindesk.com/canadas-securities-watchdog-seeks-blockchain-firms-startup-sandbox/*

[CR74] Higgins S (2017b) 'Not a Surprise': Blockchain Industry Saw SEC ICO Action Coming, July 25, Retrieved from https://www.coindesk.com/markets/2017/07/25/not-a-surprise-blockchain-industry-sawsec-ico-action-coming/*

[CR75] Higgins S (2017c) Decentralized Exchange Protocol 0x Raises $24 Million in ICO August 17, Retrieved from https://www.coindesk.com/markets/2017/08/17/decentralized-exchange-protocol-0x-raises-24-million-in-ico/*

[CR76] Hong MY, Yoon JW (2022). The impact of COVID-19 on cryptocurrency markets: a network analysis based on mutual information. PLoS ONE.

[CR77] Hornuf L, Kück T, Schwienbacher A (2021). Initial coin offerings, information disclosure, and fraud. Small Bus Econ.

[CR78] Janicke M, Jacob K (2004) Lead Markets for Environmental Innovations: A new role for the Nation State, Global Environmental Politics, 4:1, February 2004, p 31

[CR79] Johan SA, Knill A, Mauck N (2013). Determinants of sovereign wealth fund investment in private equity vs public equity. J Int Bus Stud.

[CR80] Jones C, Temouri Y (2016). The determinants of tax haven FDI. J World Bus.

[CR81] Jones J (2018) Swiss voluntary code to protect ICO investors, 10 Jan. https://icoexaminer.com/ico-news/swiss-voluntary-code-protect-ico-investors/

[CR82] Joselyn RW (1977). Designing the marketing research project.

[CR83] Kaal WA (2018). Initial coin offerings: the top 25 jurisdictions and their comparative regulatory responses. CodeX Stanf J Blockchain Law Policy.

[CR84] Karpenko OA, Blokhina TK, Chebukhanova LV (2021). The initial coin offering (ICO) process: regulation and risks. J Risk Financ Manag.

[CR85] Kasatkin S (2022). The legal content of a white paper for an ICO (initial coins offering). Inf Commun Technol L.

[CR86] Keuschnigg C, Nielsen SB (2001). Public policy for venture capital. Int Tax Public Finance.

[CR87] Keuschnigg C, Nielsen SB (2003). Tax policy, venture capital, and entrepreneurship. J Public Econ.

[CR88] Khan SAR, Godil DI, Jabbour CJC (2021). Green data analytics, blockchain technology for sustainable development, and sustainable supply chain practices: evidence from small and medium enterprises. Ann Oper Res.

[CR89] Khan SAR, Ponce P, Thomas G, Yu Z, Al-Ahmadi MS, Tanveer M (2021). Digital technologies circular economy practices and environmental policies in the era of COVID-19. Sustainability.

[CR90] Khan SAR, Razzaq A, Yu Z, Miller S (2021). Industry 4.0 and circular economy practices: a new era business strategies for environmental sustainability. Bus Strateg Environ.

[CR91] Khan SAR, Zhang Y, Sarwat S, Godil DI, Amin S, Shujaat S (2021). The role of block chain technology in circular economy practices to improve organisational performance. Int J Log Res Appl.

[CR92] Knott M, Strich F, Strunk K, Mayer A (2022). Uncovering potential barriers of using initial coin offerings to finance artistic projects. J Cult Econ.

[CR93] Konisky DM (2007). Regulatory competition and environmental enforcement: is there a race to the bottom?. Am J Polit Sci.

[CR94] Kshetri N (2015). Success of crowd-based online technology in fundraising: an institutional perspective. J Int Manag.

[CR95] Kshetri N (2017) Potential Roles of Blockchain in Fighting Poverty and Reducing Financial Exclusion in the Global South. J Glob Inf Technol Manag (20):201-204

[CR96] Kshetri N (2018). Cryptocurrencies: transparency vs privacy. Computer.

[CR97] Kshetri N (2018). Informal institutions and Internet-based equity crowdfunding. J Int Manag.

[CR98] Kshetri N (2021). Blockchain-based smart contracts to provide crop insurance for smallholder farmers in developing countries. IEEE IT Prof.

[CR99] Kshetri N (2022). Blockchain as a tool to facilitate property rights protection in the global south: lessons from India’s Andhra Pradesh state. Third World Q.

[CR100] Kshetri N, Dholakia N (2009). Professional and trade associations in a nascent and formative sector of a developing economy: a case study of the NASSCOM effect on the Indian offshoring industry. J Int Manag.

[CR101] Kshetri N (2018a) How criminals can manipulate cryptocurrency markets, conversation, May 31. https://theconversation.com/how-can-criminals-manipulate-cryptocurrency-markets-97294

[CR102] LaPorta R, Lopez-de-Silanes F, Shleifer A, Vishny RW (2002). Investor protection and corporate valuation. J Finance.

[CR103] Lee J, Li T, Shin D (2022). The wisdom of crowds in fintech*: *evidence from initial coin offerings*,* with Jongsub Lee and Donghwa Shin. Rev Corp Finance Stud.

[CR104] letstalkbitcoin.com (2014) Erik voorhees explains why some bitcoin companies are blocking American users, Oct 14. https://letstalkbitcoin.com/blog/post/erik-voorhees-explains-why-some-bitcoin-companies-are-blocking-american-users

[CR105] Li T, Kou G, Peng Y, Yu PS (2021). An integrated cluster detection, optimization, and interpretation approach for financial data. IEEE Trans Cybern.

[CR106] Li J, William M (2018) Initial coin offerings and platform building 2018 WFA, 2019 AFA, SSRN. Doi: 10.2139/ssrn.3088726.https://ssrn.com/abstract=3088726

[CR107] Lielacher A (2018) ICOs version 2.0 — what are DAICOs and will they revolutionize the ICO?, 29 May. https://bravenewcoin.com/insights/icos-version-2-0-what-are-daicos-and-will-they-revolutionize-the-ico

[CR108] Lin B, Khattak SI, Zhao B (2021). To relocate or not to relocate: a logit regression model of factors influencing corporate headquarter relocation decision in China. SAGE Open.

[CR109] Liu J, Hu M, Zhang H, Carrick J (2018). Corruption and entrepreneurship in emerging markets. Emerg Mark Finance Trade.

[CR110] MAS (2017a) MAS clarifies regulatory position on the offer of digital tokens in Singapore. Monetary Authority of Singapore. http://www.mas.gov.sg/News-and-Publications/Media-Releases/2017a/MAS-clarifies-regulatory-position-on-the-offer-of-digital-tokens-in-Singapore.aspx

[CR111] MAS (2017b) A guide to digital token offerings. Monetary Authority of Singapore, Nov 14

[CR112] MIT Technology Review (2017) Understand why Ethereum exists, and you’ll get why it’s a big&nbsp;deal, Oct 27. https://medium.com/mit-technology-review/understand-why-ethereum-exists-and-youll-get-why-it-s-a-big-deal-df6765a5805d

[CR113] MME (2018) Framework for legal and risk assessment of crypto tokens: classification of decentralized blockchain-based assets, May 1, "Block 2" Version. https://www.mme.ch/fileadmin/files/documents/180501_BCP_Framework_for_Assessment_of_Crypto_Tokens_-_Block_2.pdf

[CR114] Mallampally P, Sauvant KP (1999). Foreign direct investment in developing countries. Finance Dev.

[CR115] Marian OY (2013). Are cryptocurrencies super tax havens?. Mich l Rev.

[CR116] Marian OY (2019) Blockchain havens and the need for their internationally-coordinated regulation. N.C.J.L. &&nbsp;Tech, 20. https://ssrn.com/abstract=3357168

[CR117] Marinoff N (2018) China blocks access to over 120 offshore digital currency exchanges. https://bitcoinmagazine.com/articles/china-blocks-access-over-120-offshore-digital-currency-exchanges/

[CR118] Matonis J (2014) Beyond New York: What Lies Ahead for Bitcoin, July 24, https://www.coindesk.com/markets/2014/07/24/beyond-new-york-what-lies-ahead-for-bitcoin/

[CR119] Megginson WL, Weiss KA (1991). Venture capitalist certification in initial public offerings. J Finance.

[CR120] Meuleman M, Maeseneire WD (2012). Do R&D subsidies affect SMEs' access to external financing?. Res Policy.

[CR121] Milano A (2018) Bermuda drafting ICO-friendly legislation to draw crypto businesses, Mar 20. https://www.coindesk.com/bermuda-drafting-ico-friendly-legislation-to-draw-crypto-businesses

[CR122] Miles MB, Huberman AM (1994). Qualitative data analysis.

[CR123] Miller D, Friesen PH (1983). Successful and unsuccessful phases of the corporate life cycle. Organ Stud.

[CR124] Mohamadi A, Peltonen J, Wincent J (2017). Government efficiency and corruption: a country-level study with implications for entrepreneurship. J Bus Ventur Insights.

[CR125] Mollick E (2014). The dynamics of crowdfunding: an exploratory study. J Bus Ventur.

[CR126] Momtaz PP (2020). Initial coin offerings. PLoS ONE.

[CR127] Morris D (2017) SEC files fraud charges against two ICOs, Oct 04. http://fortune.com/2017/10/01/sec-ico-fraud-charges/

[CR128] Murtinu S, Scalera VG (2016). Sovereign wealth funds’ internationalization strategies: the use of investment vehicles. J Int Manag.

[CR129] Müller L, Meyer SD, Gschwend C, Henschel P (2017) Conceptual framework for legal & risk assessment of blockchain crypto property (BCP), MME, Genesis Version of 26.09.17

[CR130] North DC (1990). Institutions, institutional change and economic performance.

[CR131] newsbtc.com (2016a) Switzerland to introduce new banking license for fintech startups, Nov 03. https://www.newsbtc.com/2016a/11/03/switzerland-introduce-new-banking-license-fintech-startups/

[CR132] newsbtc.com (2016b) BTC remains the only viable option to avoid capital controls in China. https://www.newsbtc.com/2016b/12/19/bitcoin-remains-viable-option-avoid-capital-controls-china/

[CR133] newsbtc.com (2017) Mauritius plans to create an Ethereum Island. https://www.newsbtc.com/2017/07/16/mauritius-plans-create-ethereum-island/

[CR134] O'Leary RR (2017a) Putin advisor announces new blockchain advocacy group, Aug 31. https://www.coindesk.com/russia-announces-new-blockchain-association

[CR135] O'Leary RR (2017b) South Korean regulator issues ICO ban. https://www.coindesk.com/south-korean-regulator-issues-ico-ban

[CR136] Parker L (2017) Crypto valley association building a concentrated blockchain ecosystem in Switzerland, Brave New Coin. https://bravenewcoin.com/insights/crypto-valley-association-building-a-concentrated-blockchain-ecosystem-in-switzerland

[CR137] Parker L (2016) Block chain tech companies focus on the Million in ICO August 17, Retrieved from https://www.coindesk.com/markets/2017/08/17/decentralized-exchange-protocol-0x-raises-24-million-in-ico/.

[CR138] Pilarowski G, Yue L (2017) China bans initial coin offerings and cryptocurrency trading platforms, China Regulation Watch, 21 Sept 2017. http://www.pillarlegalpc.com/en/news/2017/09/21/china-bans-initial-coin-offerings-and-cryptocurrency-trading-platforms/

[CR139] Porter ME (1990). The competitive advantage of nations.

[CR140] Porter ME (1996). What is strategy?. Harv Bus Rev.

[CR141] Powell WW (1993) The social construction of an organizational field: the case of biotechnology. Paper presented at the Warwick–Venice Workshop on perspectives on strategic change, University of Warwick

[CR142] Porter ME, Van der Linde C (1995) Green and Competitive Ending the Stalemate. Harvard Business Rev 73, 120-134.

[CR143] Pozzi D (2019) ICO Market 2018 vs 2017: trends, capitalization, localization, industries, success rate, Jan 5. https://cointelegraph.com/news/ico-market-2018-vs-2017-trends-capitalization-localization-industries-success-rate

[CR144] RESSOS (2018) How to do an initial coin offering (ICO) in Singapore July 6, Ressos Legal Pte Ltd. https://ressos.com/downloads/Ressos%20-%20How%20to%20do%20an%20ICO%20in%20Singapore.pdf

[CR145] Read R (2001) Growth, economic development and structural transition in small vulnerable states, WIDER Discussion Paper, No. 2001/59, The United Nations University World Institute for Development Economics Research (UNU-WIDER), Helsinki

[CR146] Reeves C (2017) Blockchain and cryptocurrency are the future of offshore banking. http://premieroffshore.com/blockchain-cryptocurrency-future-offshore-banking/

[CR147] Reutzel B (2018) Puerto Rico aims to attract blockchain startups with new council, Mar 15. https://www.coindesk.com/puerto-rico-aims-attract-blockchain-startups-new-council

[CR148] Da Rin M, Nicodano G, Sembenelli A (2006). Public policy and the creation of active venture capital markets. J Public Econ.

[CR149] Rizzo P (2014) Crypto 2.0 industry dismisses sec crackdown rumors, Oct 28. https://www.coindesk.com/crypto-2-0-companies-rebuff-sec-crackdown-rumors

[CR150] SEC (2017) SEC issues investigative report concluding DAO tokens, a digital asset, were securities, the securities and exchange commission, July 25. https://www.sec.gov/news/press-release/2017-131

[CR151] Scaggs A (2018) ICO regulator anger translator, January 23, https://www.ft.com/content/50e2d60c-c4a1-3d30-af91-c154647e13d5

[CR152] Scott WR (1992). Organizations: rational, natural and open systems.

[CR153] Scott WR (1995). Institutions and organizations.

[CR154] Seo MG, Creed WED (2002). Institutional contradictions, praxis, and institutional change: a dialectical perspective. Acad Manag Rev.

[CR155] Setser BW (2008) Norway was against Iceland before it was for Iceland, May 17. https://www.cfr.org/blog/norway-was-against-iceland-it-was-iceland

[CR156] Shaxson N (2016) Five myths about tax havens. https://www.washingtonpost.com/opinions/five-myths-about-tax-havens/2016/04/15/76d001d2-0255-11e6-b823-707c79ce3504_story.html. Accessed 18 Sept 2017

[CR157] Shin L (2017) After contact by SEC, protostarr token shuts down post-ICO, will refund investors. Sept 06. https://www.forbes.com/sites/laurashin/2017/09/01/after-contact-by-sec-protostarr-token-shuts-down-post-ico-will-refund-investors/#7496a499192e

[CR158] Sigle H (2017) Regulation of initial coin offerings (ICO) in Germany. https://www.lexology.com/library/detail.aspx?g=86bd9cd2-144c-4ddd-b65e-bdac2099b115. Accessed 10 Sept 2017

[CR159] Simpson I (2017) ICO central: why Switzerland will remain crypto valley, Oct 6. https://www.coindesk.com/ico-central-switzerland-will-remain-crypto-valley

[CR160] Slemrod J, Wilson JD (2009). Tax competition with parasitic tax havens. J Public Econ.

[CR161] Spigel B (2017). The relational organization of entrepreneurial ecosystems. Entrep Theory Pract.

[CR162] Stanley A (2017a) ConsenSys, nation of mauritius in talks to create 'Ethereum Island'. https://www.coindesk.com/consensys-nation-mauritius-talks-create-ethereum-island/

[CR163] Stanley A (2017b) Mauritius: the tropical paradise looking to become a blockchain hub. https://www.coindesk.com/mauritius-the-tropical-paradise-looking-to-become-a-blockchain-hub

[CR164] Stanley A (2017c) ICO ban? canada's regulators are giving one token sale a big break, 18 Sept. https://www.coindesk.com/ico-ban-canadas-regulators-giving-one-token-sale-big-break/

[CR165] Stenholm P, Acs ZJ, Wuebker R (2013) Exploring country-level institutional arrangements on the rate and type of entrepreneurial activity. J Business Ventur 8(1):176–193

[CR166] Sundararajan S (2017) Singapore’s central bank outlines when ICOs are and aren’t securities, 15 Nov. https://www.coindesk.com/singapores-central-bank-outlines-icos-arent-securities

[CR167] Sutton RI, Staw BM (1995). What theory is not. Adm Sci Q.

[CR168] Swartz L (2022). Theorizing the 2017 blockchain ICO bubble as a network scam. New Media Soc.

[CR169] swissinfo.ch (2018) Swiss financial watchdog publishes ICO guidelines, 16 Feb. https://www.swissinfo.ch/eng/more-clarity_swiss-financial-watchdog-publishes-ico-guidelines/43906332

[CR170] Szabo N (1994). Smart contracts. Unpublished manuscript

[CR171] Thomas DR (2006). A general inductive approach for analyzing qualitative evaluation. Data Am J Eval.

[CR172] Tian C (2017a) China's new fundraising rules could lead to ICO investigations. https://www.coindesk.com/chinas-new-fundraising-rules-lead-ico-investigations/

[CR173] Tian C (2017b) Report: China's regulators close to taking action against ICOs. https://www.coindesk.com/report-chinas-regulators-close-taking-action-icos/

[CR174] Tolbert PS, Zucker LG, Clegg S, Hardy C, Nord WR (1996). The institutionalization of institutional theory. Handbook of organization studies.

[CR175] Uribe-Toril J, Ruiz-Real JL, Ceresia F, de Pablo Valenciano J (2019). Corruption and entrepreneurship: a bibliometric analysis. J Leg Ethical Regul Issues.

[CR176] van Oosterhout A (2021) ICOs ‘will disappear in 2020’ as data shows 95% funding decline, 1 Oct. https://bitcoinist.com/icos-will-disappear-in-2020-as-data-shows-95-funding-decline/

[CR177] Vigna P (2014) BitBeat: bitcoin bulls get a buying opp; Bitcoin anarchists declare their independence. Wall Str J. http://blogs.wsj.com/moneybeat/2014/08/15/bitbeat-bitcoin-bulls-get-a-buying-opp-bitcoin-anarchists-declare-their-independence/

[CR178] Vogel SK (1996). Freer markets, more rules: regulatory reform in advanced industrial countries.

[CR179] Volden C, Ting M, Carpenter D (2008). A formal model of learning and policy diffusion. Am Pol Sci Rev.

[CR180] WB (World Bank) (2019) Doing business 2019. https://www.doingbusiness.org/en/reports/global-reports/doing-business-2019

[CR181] WEF (2015) Deep shift technology tipping points and societal impact survey report. World Economic Forum. http://www3.weforum.org/docs/WEF_GAC15_Technological_Tipping_Points_report_2015.pdf

[CR182] Waters R (2017) To coin a craze: Silicon Valley’s cryptocurrency boom, September 13 https://www.ft.com/content/2b0d8926-96d9-11e7-b83c-9588e51488a0

[CR183] Weber R (2012). Evaluating and developing theories in the information systems discipline. J Assoc Inf Syst.

[CR184] Werder B (2017) Federal council initiates consultation on new fintech regulations. The Federal Council. https://www.admin.ch/gov/en/start/documentation/media-releases.msg-id-65476.html

[CR185] Whetten DA, Partington D (2002). Modeling-as-theorizing: a systematic methodology for theory development. Essential skills for management research.

[CR186] Wilson F (2017) What happened in 2017, AVC, 31 Dec. https://avc.com/2017/12/what-happened-in-2017/

[CR187] Wilson T (2020) Where ‘DeFi’ crypto users see sure bets during COVID, critics see unregulated risks, 27 Aug. https://www.insurancejournal.com/news/international/2020/08/27/580591.htm

[CR188] Wolfson R (2017) Purchasing property online: the revolutionary way ukraine uses blockchain for real estate. https://www.huffingtonpost.com/entry/purchasing-property-online-the-revolutionary-way-ukraine_us_59933fe7e4b0afd94eb3f565

[CR189] Xu M, Chen X, Kou G (2019). A systematic review of blockchain. Finance Innov.

[CR190] Yaga D, Mell P, Roby N, Scarfone K (2018) Blockchain technology overview, National Institute of Standards and Technology Internal Report (NISTIR) 8202

[CR191] Yashu G (2017) Panama's blockchain-based prime-ex perpetual announces pre-ICO sale round. http://www.newsbtc.com/2017/09/07/panamas-blockchain-based-prime-ex-perpetual-announces-pre-ico-sale-round/

[CR192] Yu Z, Khan SAR, Umar M (2022). Circular economy practices and industry 4.0 technologies: a strategic move of automobile industry. Bus Strategy Environ.

[CR193] Yu E, Ostroff C (2021) Chinese police arrest 1,100 people for money laundering with cryptocurrencies, 10 June. https://www.wsj.com/articles/chinese-police-arrest-1-100-people-for-money-laundering-with-cryptocurrencies-11623332776

[CR194] Zagone R (2017) Ripple supports efforts to drive thoughtful regulation, 22 Feb 2017. https://ripple.com/ko/insights/ripple-supports-efforts-drive-thoughtful-regulation/

[CR195] Zetsche DA, Buckley RP, Arner DW, Barberis JN (2018). From fintech to techfin: the regulatory challenges of data-driven finance. Nyujl Bus.

[CR196] Zhao W (2018a) Proposed US task force would tackle crypto use in terrorism financing, 17 Jan. https://www.coindesk.com/proposed-us-task-force-would-tackle-crypto-use-in-terrorism-financing

[CR197] Zhao W (2018b) China’s crypto exchanges didn’t just survive—they’re thriving. https://www.coindesk.com/chinas-crypto-exchanges-didnt-just-survive-theyre-thriving

[CR198] Zhao W (2018c) Coinbase tells 13,000 users it’s sending their data to the IRS. https://www.coindesk.com/coinbase-tells-13000-users-its-sending-their-data-to-the-irs

[CR199] Zollo M, Winter SG (2002). Deliberate learning and the evolution of dynamic capabilities. Organ Sci.

[CR200] Zucman G (2015). The scourge of tax havens. The hidden wealth of Nations.

